# Influence of Aza-Substitution on Molecular Structure, Spectral and Electronic Properties of *t*-Butylphenyl Substituted Vanadyl Complexes

**DOI:** 10.3390/ijms27020606

**Published:** 2026-01-07

**Authors:** Daniil N. Finogenov, Alexander E. Pogonin, Yuriy A. Zhabanov, Ksenia V. Ksenofontova, Dominika Yu. Parfyonova, Alexey V. Eroshin, Pavel A. Stuzhin

**Affiliations:** Research Institute of Chemistry of Macroheterocyclic Compounds, Ivanovo State University of Chemistry and Technology, Sheremetevskiy Av. 7, 153000 Ivanovo, Russia; pogoninalexander@mail.ru (A.E.P.); kvk@isuct.ru (K.V.K.); dominikaparfenova633@gmail.com (D.Y.P.); alexey.yeroshin@gmail.com (A.V.E.); stuzhin@isuct.ru (P.A.S.)

**Keywords:** phthalocyanines, tetrapyrazinoporphyrazines, electrochemistry, basic properties, DFT calculations

## Abstract

Vanadyl octa-(4-*tert*-butylphenyl)phthalocyanine (**VOPc(*t*-BuPh)_8_**) and vanadyl octa-(4-*tert*-butylphenyl)tetrapyrazinoporphyrazine (**VOTPyzPz(*t*-BuPh)_8_**) complexes were synthesized for the first time and confirmed by IR and UV-Vis spectroscopy and MALDI-TOF spectrometry. The method of synthesis of their precursors, 4,5-bis(4-*tert*-butylphenyl)phthalonitrile (**(*t*-BuPh)_2_PN**) and 5,6-bis(4-*tert*-butylphenyl)pyrazine-2,3-dicarbonitrile (**(*t*-BuPh)_2_PDC**), was modified, resulting in higher yields. For the vanadyl complexes, the basic properties were studied, and it was found that the red shift in the Q band in the first protonation step is approximately two times greater than that of previously known complexes. An electrochemical study showed the influence of aza-substitution on the redox properties and on the energies of the frontier orbitals of all the compounds presented. For all four considered compounds, quantum chemical calculations of the molecular structure, IR spectra, and electronic absorption spectra were carried out using density functional theory (DFT) and time-dependent density functional theory (TDDFT and simplified sTDDFT) approaches. According to the DFT calculations, vanadyl macrocyclic complexes have dome-shaped distorted structures. Experimental and theoretical IR and electronic absorption spectra were compared and interpreted.

## 1. Introduction

Phthalocyanines (**Pc**s) and their aza-analogs, tetrapyrazinoporphyrazines (**TPyzPz**s), are promising porphyrinoids in various applications such as dyes, catalysts, and materials for optical, electronic, and photoelectronic devices [1,2,3,4,5,6,7,8,9,10,11]. Among such macrocycles, vanadium complexes are of particular interest due to the paramagnetic properties of the central metal atom, as well as to the presence of an axial ligand that promotes packing in crystals. Research by Hadt and colleagues has shown that vanadyl and titanyl phthalocyanines could be promising options for application in quantum computing [12,13]. Nemykin et al. [14] have also noted that tetrapyrazinoporphyrazines could be successfully used in this field. Moreover, vanadyl complexes of phthalocyanines and their analogs have been extensively studied, since they have the potential to form compounds with coupling between spins [14,15,16,17,18,19,20].

The synthesis of alkyl-substituted aza-analogs of phthalocyanines and naphthalocyanines [21,22,23], which can be dissolved in common organic solvents such as chloroform, as well as compounds with isocyanides [17] or axial dialkylsiloxane ligands [18], has already been carried out. At the same time, it is known that the addition of aryl substituents to phthalocyanines, naphthalocyanines, and their aza-analogs significantly increases their solubility in most organic solvents due to disruption of the planar structure of these molecules [24]. The combination of these types of substitution at the periphery of macrocyclic core provides excellent solubility.

Derivatives of phthalonitrile (**PN**, 1,2-dicyanobenzene, phthalodinitrile, *o*-phthalonitrile) and pyrazine-2,3-dicarbonitrile (**PDC**, 2,3-dicyanopyrazine, 2,3-pyrazinedicarbonitrile) are used as precursors for the production of the macroheterocycles mentioned above. Despite the large number of known phthalonitriles, the study of their structures, vibrational spectra, and electronic spectra has received much less attention than the corresponding macrocycles. However, we should not forget that the conformational properties of initial phthalonitriles can influence the structure of macrocycles produced from them [25,26,27].

In this work, we synthesized new vanadyl octa-(4-*tert*-butylphenyl)phthalocyanine (**VOPc(*t*-BuPh)_8_**) and vanadyl octa-(4-*tert*-butylphenyl)tetrapyrazinoporphyrazine (**VOTPyzPz(*t*-BuPh)_8_**) complexes and slightly modified the existing method for obtaining precursors—4,5-bis(4-*tert*-butylphenyl)phthalonitrile (**(*t*-BuPh)_2_PN**) and 5,6-bis(4-*tert*-butylphenyl)pyrazine-2,3-dicarbonitrile (**(*t*-BuPh)_2_PDC**). The molecular structurse, infrared (IR) and ultraviolet–visible (UV-Vis) spectra of the compounds were also studied.

## 2. Results and Discussion

### 2.1. Synthesis

**Scheme 1:**
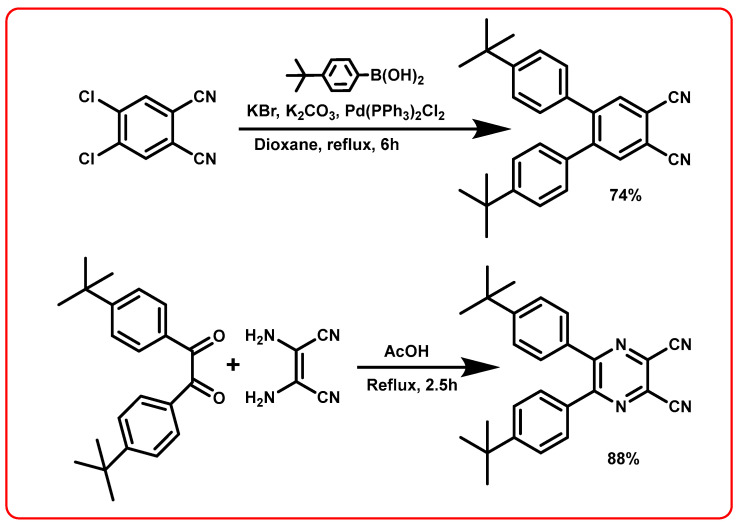
Synthesis of dinitrile precursors **(*t*-BuPh)_2_PN** and **(*t*-BuPh)_2_PDC**.

Firstly, we optimized previously reported synthetic methods of **(*t*-BuPh)_2_PN** [28,29] and **(*t*-BuPh)_2_PDC** [30] (Scheme 1). **(*t*-BuPh)_2_PN** was obtained in a higher yield compared to the results reported in previously published synthesis methods [28]. It was facilitated by the use of a procedure known for **Ph_2_PN** [31]. After completion of the reaction, purification by precipitation or flash chromatography yielded a greenish-gray product containing trace amounts of phthalocyanine. Obtaining a white powder of **(*t*-BuPh)_2_PN** is possible with additional sublimation at 250 °C using a water pump.

The synthesis of **(*t*-BuPh)_2_PDC** was slightly modified at the purification stage. After boiling in acetic acid, the resulting mixture was dried under vacuum on a rotary evaporator and purified by a silica gel flash column with DCM as the eluent to give a pale yellow powder with an 88% yield. This value is significantly higher than the 34% yield published earlier [30]. The change in purification method was undertaken because the previous separation [30] was not optimized, and boiling with activated charcoal led to significant product losses.

**Scheme 2:**
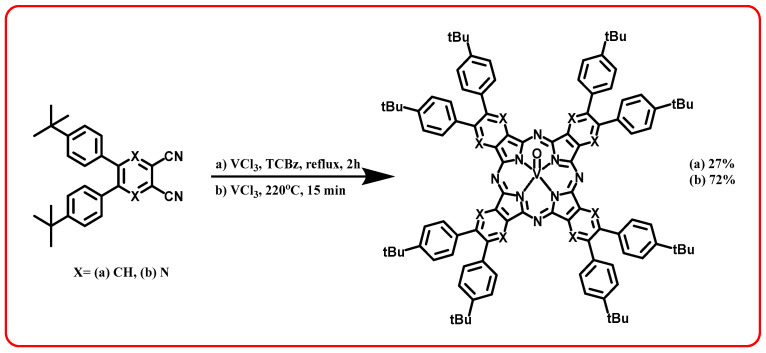
Synthesis of (a) **VOPc(*t*-BuPh)_8_** and (b) **VOTPyzPz(*t*-BuPh)_8_**.

In the case of vanadyl complexes, different synthetic methods (Scheme 2) were applied for **(*t*-BuPh)_2_PN** and **(*t*-BuPh)_2_PDC**. For **VOTPyzPz(*t*-BuPh)_8_**, melting of the corresponding dinitrile with vanadium chloride at 220 °C gave the target complex in good yield. On the other hand, melting of **(*t*-BuPh)_2_PN** with VCl_3_ under the same conditions did not yield a phthalocyanine, even when the temperature was increased to 265 °C. Tetramerization in a high-boiling solvent led to the formation of **VOPc(*t*-BuPh)_8_**, but the yield was much lower than in the case of **VOTPyzPz(*t*-BuPh)_8_** (27% vs. 72%, respectively).

### 2.2. Basic Properties

**Figure 1:**
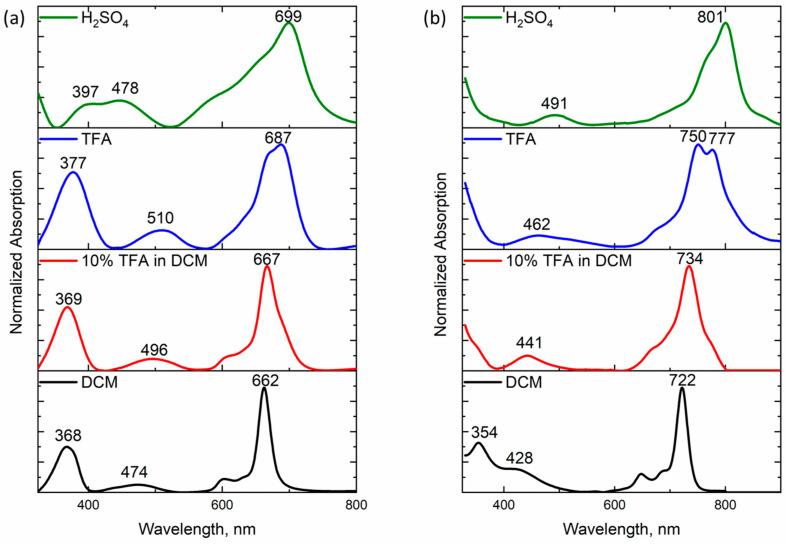
UV-Vis spectra of (**a**) **VOTPyzPz(*t*-BuPh)_8_** and (**b**) **VOPc(*t*-BuPh)_8_** in solutions with different acidity.

The spectral behavior of the considered vanadyl complexes in various acidic media were studied (Figure 1). The presence of nitrogen atoms at *meso*-positions in both complexes and in the pyrazine rings in the case of **VOTPyzPz(*t*-BuPh)_8_** as basic centers provides the possibility to study their protonation, which is typical for metal phthalocyanines and their analogs [32,33,34,35].

With increasing acidity of the medium, sequential protonation of meso-nitrogen occurs, which leads to a characteristic splitting and a bathochromic shift in the Q band maximum. It was shown that the shape of the Q band and the bathochromic shift in its long-wave maximum are unique for each stage of protonation [35]. Previously, our group showed that substituents of an acceptor nature have a slight effect on shifts in absorption spectra in the case of phthalocyanines (see Table 1). Herein, for the molecule with electron-donating substituents **VOPc(*t*-BuPh)_8_**, we can see another influence. For this phthalocyanine, the first protonation step has a 980 cm^−1^ shift in the Q band from 722 nm to 777 nm (Table 1), which is almost twice as large as that of the corresponding **MPcCl_8_** and **MPc(*t*-Bu)_4_** derivatives of group 13 complexes [32,33]. This may be due to the presence of more electron-rich fragments at the periphery of the molecule; additionally, protonation of the axial oxygen at the vanadium atom is observed, as seen in Figure 1, with addition of 10% trifluoroacetic acid (TFA), manifested as a slight shift and broadening in the Q band. The second protonation step can only be detected in pure sulfuric acid, and the overall shift in the Q band is approximately 79 nm (1366 cm^−1^) from 722 nm to 801 nm (Table 1). In addition, protonation also affects the position of the charge-transfer (CT) band, which shifts by 34 nm in the case of the first protonation step and by 63 nm in the case of the second one (see Figure 1). On the other hand, **VOTPyzPz(*t*-BuPh)_8_** demonstrates behavior similar to **MTPyzPzCl_8_** [34]. The first protonation step is observed at 687 nm of the Q band, with a 25 nm (840 cm^−1^) shift (see Figure 1). Subsequent acidification of the solution by pure H_2_SO_4_ leads to protonation of the pyrazine nitrogen atoms, which can be detected by broadening of the Q band and splitting of the CT band (Figure 1).

### 2.3. Electrochemistry

**Figure 2:**
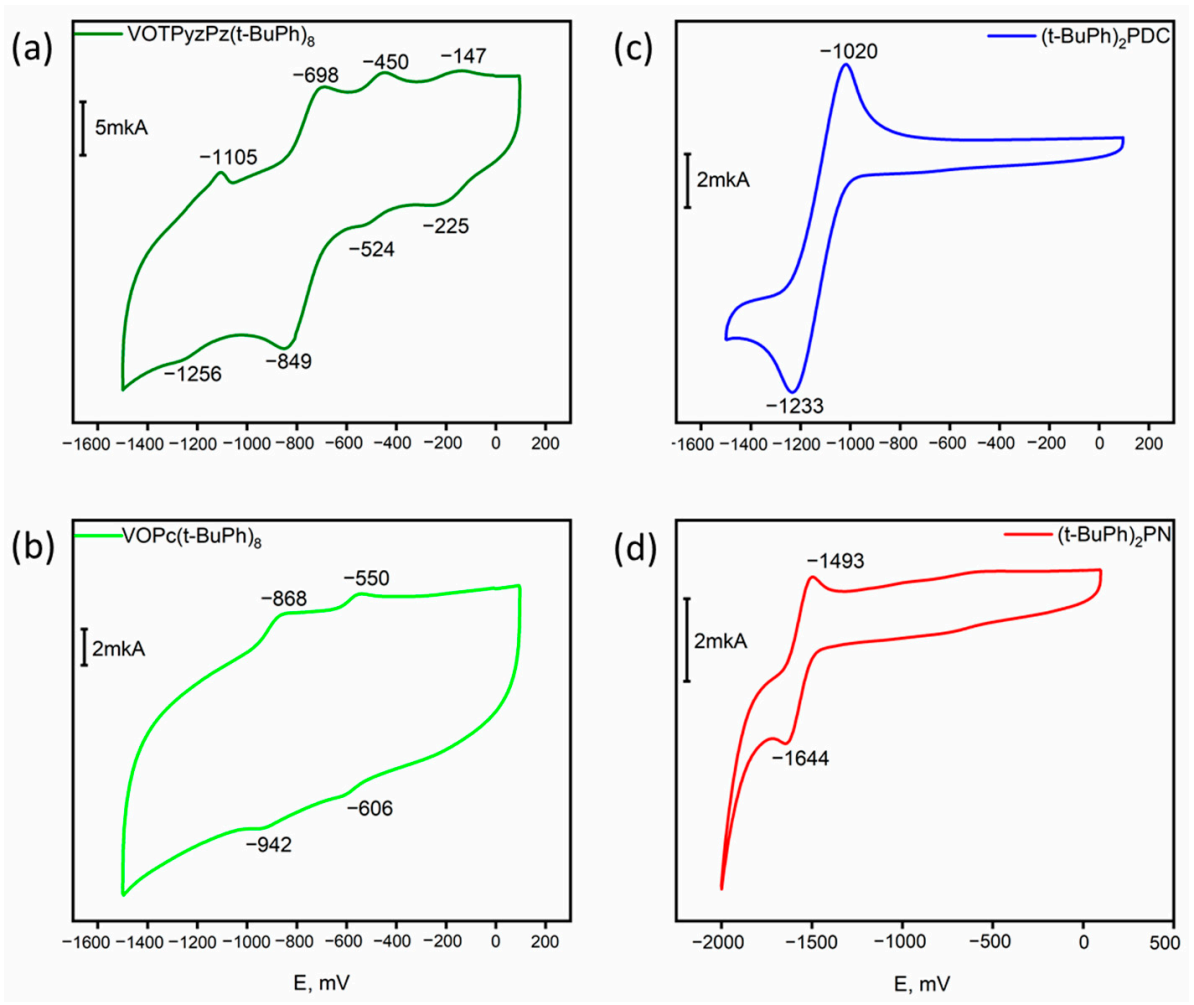
Cyclic voltammograms for (**a**) **VOTPyzPz(*t*-BuPh)_8_**, (**b**) **VOPc(*t*-BuPh)_8_**, (**c**) **(*t*-BuPh)_2_PDC**, and (**d**) ***(t*-BuPh)_2_PN** measured in DCM (100 mV/s scan rate, 0.1 M TBAP).

Cyclic voltammograms for studied dinitriles and macroheterocycles are shown in Figure 2, Table 2 contains the determined values of reduction potentials for the studied compounds in comparison with the corresponding values for related vanadyl complexes—**VOPc(NaphtO)_4_**, **VOPc(*t*-Bu)_2_C_6_H_3_O)_4_**, **VOPc(C_8_H_17_O)_4_**, **VOTPyzPzCl_8_**, **VOTTDPz**, **VOPc**, and **VOTBP**. For the dinitriles, only one reduction process was observed. In this case, the influence of aza-substitution manifests as a shift in the first reduction potential toward 0 (Figure 2c,d). A similar tendency is observed when comparing the **VOTPyzPz(*t*-BuPh)_8_** and **VOPc(*t*-BuPh)_8_** complexes.

A pyrazine-annulated macrocycle can be easily reduced in comparison with its benzo-annulated analogs by ~400 mV. This phenomenon is related to the electron-deficient nature of the pyrazine rings and leads to a decrease in the energy of the lowest unoccupied molecular orbital (LUMO). On the other hand, peripheral substitution in benzene rings appears to have little effect on the values of the reduction potentials in the case of Pcs. Thus, **VOTPyzPz(*t*-BuPh)_8_** and **VOTPyzPzCl_8_** have similar first reduction potentials.

Phthalocyanine complexes show similar behavior of the first reduction potential, with values ranging from −510 to −640 mV. In this row, **VOPc(*t*-Bu)_2_C_6_H_3_O)_4_** has the highest value of *E*_1/2_*,* but in this case the impact of substituents is quite low. These complexes also possess a greater acceptor nature than **VOTBP** (vanadyl tetrabenzoporphyrin). **VOPc(*t*-BuPh)_8_** has the highest second reduction potential in comparison with other considered complexes, which can provide easy formation of dianions with different reductants for single-molecule magnets [18].

The values of LUMO energies were estimated using the data of electrochemical experiments by the empirical Equation (1) [41].

(1) $$E_{LUMO}=-1.19\cdot (E_{1/2\;red}^{1}-E_{1/2}(Fc/{Fc}^{+}))-4.78$$

where *E_LUMO_* is the energy of the LUMO, *E*^1^_1/2_ *_red_* is the first reduction potential, *E*_1/2_(*Fc*/*Fc*^+^) = 0.507 V is the redox potential of the reference *Fc*/*Fc*^+^ couple, and −1.19 ± 0.08 eV/V and −4.78 ± 0.17 eV are the slope and intercept of the empirical equation [37].

The UV-Vis optical gaps (*OE_G_*) were found using the absorption onset edge method by Equation (2) [41,42,43].

(2) $${OE}_{G}=\frac{1240}{\lambda_{edge}}$$

where *OE_G_* is the UV-Vis optical gap in eV, and *λ_edge_* is the onset wavelength (nm) derived at a low-energy absorption band using the online version of the program “0nset” [44].

The energies of the highest occupied molecular orbitals (HOMOs) were estimated using the experimental data of UV-Vis spectrometry and CVA by Equation (3).

(3) $$E_{HOMO}=E_{LUMO}-{OE}_{G}$$

where *E_LUMO_* was calculated by Equation (1), and *OE_G_* was calculated by Equation (2).

The experimentally and theoretically estimated values of energy gaps are presented in Table 3. It is also necessary to note the different nature of the energy values determined using different approaches, which is described in detail in [42].

### 2.4. Molecular Structure

**Figure 3:**
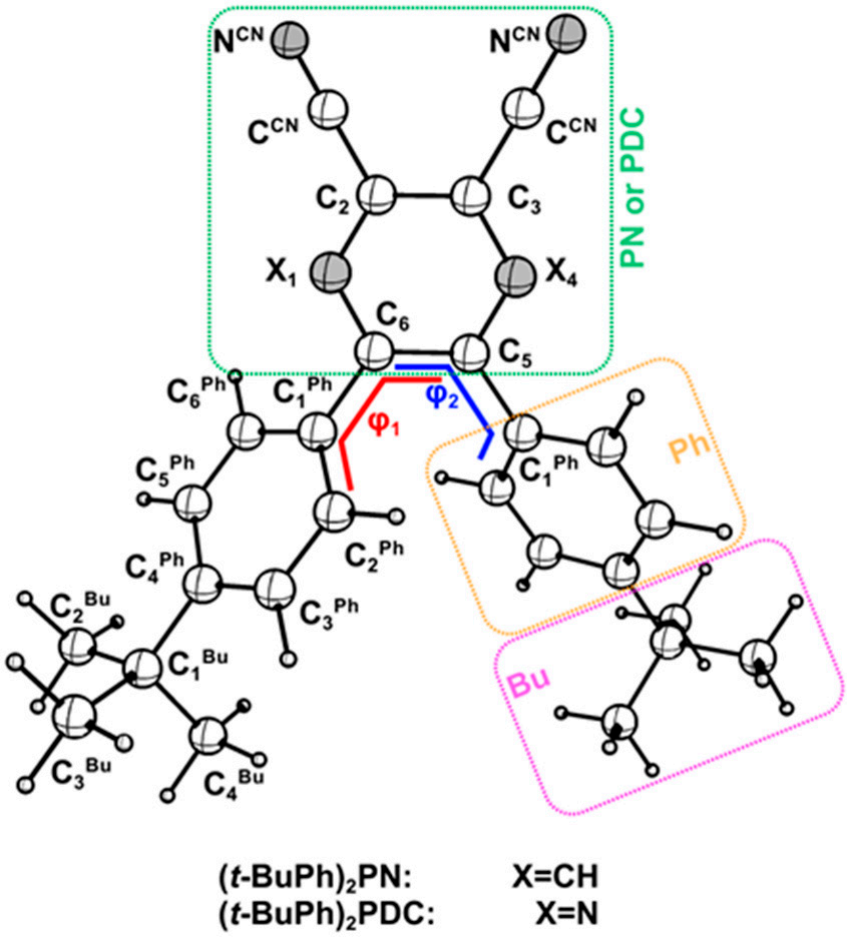
Molecular structure of **(*t*-BuPh)_2_PN** (X = CH) and **(*t*-BuPh)_2_PDC** (X = N). Atom numbering was performed by analogy with [45]. Cartesian coordinates of the structures are given in Supplementary Materials.

According to PBE0-D3BJ/def2-TZVP calculations, **PN** and **PDC** have planar structures with C_2v_ symmetry. Under **PN** → **(*t*-BuPh)_2_PN** and **PDC** → **(*t*-BuPh)_2_PDC**, substitution of hydrogen atoms with bulky *t*-BuPh groups leads to a significant increase in C_5_-C_6_ distance by 0.02 and 0.03 Å, respectively (Table 4, Figure 3). In the cases of **(*t*-BuPh)_2_PN** and **(*t*-BuPh)_2_PDC**, the presence of *t*-BuPh substituents in adjacent positions is characterized by steric repulsions in the structure; therefore, the phenyl groups are rotated relative to the molecule backbone. Neighboring *t*-BuPh groups are oriented in a “quasi-parallel manner” as in similar dinitriles [45]. These structures of **(*t*-BuPh)_2_PN** and **(*t*-BuPh)_2_PDC** are characterized by the point symmetry group C_2_. The rotation angles of the two phenyl rings (φ_1_ = φ_2_) are 49.5° and 37.3° for **(*t*-BuPh)_2_PN** and **(*t*-BuPh)_2_PDC**, respectively. The rotation of the phenyl rings in these structures is energetically hindered (Figure S11) due to their close proximity to each other. C_s_-conformations of **(*t*-BuPh)_2_PN** and **(*t*-BuPh)_2_PDC** with mirrored orientations of neighboring *t*-BuPh-groups (φ_1_ = −φ_2_) are energetically unfavorable by ~15 and ~28 kJ∙mol^−1^, respectively.

The noticeable mutual influence of *t*-BuPh groups on each other in both **(*t*-BuPh)_2_PN** and **(*t*-BuPh)_2_PDC** is also proven by the fact that, in the presence of only one *t*-BuPh group in the structure, the rotation angles φ decrease by more than 10° (35.5° for ***t*-BuPhPN** and 16.7° for ***t*-BuPhPDC**). In general, this rotation angle φ may be important, since it affects the degree of π-delocalization, which in turn significantly determines the nature of the electronic transitions and the corresponding absorption spectra (e.g., Figure S12 for ***t*-BuPhPN** and ***t*-BuPhPDC**). The barriers to rotation of *t*-BuPh-group in ***t*-BuPhPN** and ***t*-BuPhPDC** are ~12 and ~23 kJ∙mol^−1^, respectively (Figure S13).

The considered vanadyl complexes are characterized by a dome-shaped distortion of the macroheterocyclic skeleton. According to gas electron diffraction and X-ray diffraction methods, the vanadium atoms in **VOPc** are out of the coordination plane defined by the four N_p_ atoms by 0.576(14) Å [46] and 0.575(1) Å [46,47], respectively. According to ROP-BE0-GD3BJ/def2-TZVP calculations, the displacements (h) of the vanadium atom from the coordination plane in **VOPc(*t*-BuPh)_8_** and **VOTPyzPz(*t*-BuPh)_8_** are 0.587 Å and 0.575 Å, respectively. The slightly larger deviation of the V atom from the coordination plane in the case of **VOPc(*t*-BuPh)_8_** can be explained by the smaller size of the coordination plane of phthalocyanine compared to tetrapyrazinoporphyrazine (r_e_(N_p_···N_p_) in **[Pc]^2^**^−^ and **[TPyzPz]^2−^** are 3.932 Å and 3.965 Å, respectively). Neighboring *t*-BuPh groups are oriented in a “quasi-parallel manner” similar to that of the corresponding precursors (φ ≈ 50 and 40°). At the same time, the **VOPc(*t*-BuPh)_8_** and **VOTPyzPz(*t*-BuPh)_8_** molecules are characterized by conformational multiformity caused by the possibility of different orientations of the four pairs of phenyl groups. However, the energy differences between these structures are small (e.g., according to UPBE0-GD3BJ/def2-SVP calculations, the C_4_ and C_2v_ structures (see Figure S14) differ from each other by less than 1.5 kJ·mol^−1^). Subsequently, we considered theoretical spectra only for structures with the C_4_ point group. The influence of *tert*-butylphenyl groups on the structure of the macroheterocyclic skeleton is limited to some changes in the X_γ_-C_δ_ and C_δ_-C_δ_ distances (Table 5).

**Table 5:** Selected structural parameters ^a^ of vanadyl complexes by ROPBE0-D3BJ/def2-TZVP/gas calculations.

	VOPc	VOPc(*t*-BuPh)_8_	VOTPyzPz	VOTPyzPz(*t*-BuPh)_8_
Symmetry	C_4v_	C_4_	C_4v_	C_4_
V-O	1.562	1.562	1.558	1.560
h ^b^	0.586	0.587	0.572	0.575
V-N_p_	2.038	2.038	2.045	2.044
N_p_-C_α_	1.367	1.368	1.368	1.370
N_m_-C_α_	1.315	1.315	1.310	1.311
C_α_-C_β_	1.448	1.447	1.453	1.452
C_β_-C_β_	1.398	1.396	1.396	1.391
C_β_-X_γ_	1.387	1.384	1.327	1.323
X_γ_-C_δ_	1.384	1.391	1.323	1.328
C_δ_-C_δ_	1.401	1.422	1.407	1.436
C_δ_-C^Ph^	-	1.478	-	1.474
φ_1_ ≈ φ_2_	-	50.0	-	39.9

^a^ internuclear distances r_e_—in Å, valence angles—in °; designations of atoms and angles are shown in Figure 4. ^b^ h—the distance between a vanadium atom and the center of four N_p_ atoms. Cartesian coordinates of the structures are given in Supplementary Materials.

**Figure 4:**
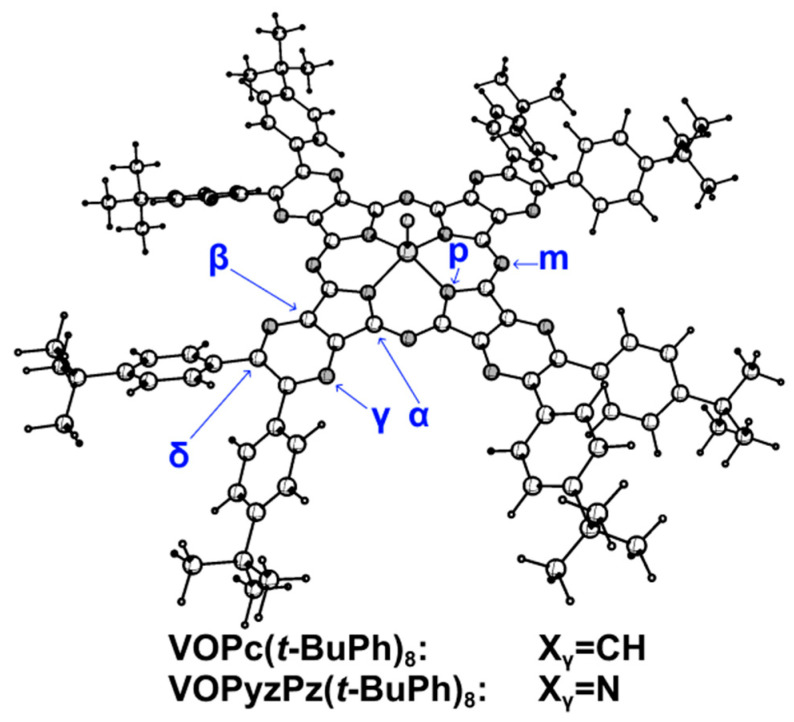
Molecular structure of **VOPc(*t*-BuPh)_8_** (Xγ = CH) and **VOTPyzPz(*t*-BuPh)_8_** (Xγ = N).

### 2.5. Electronic Structure

**Figure 5:**
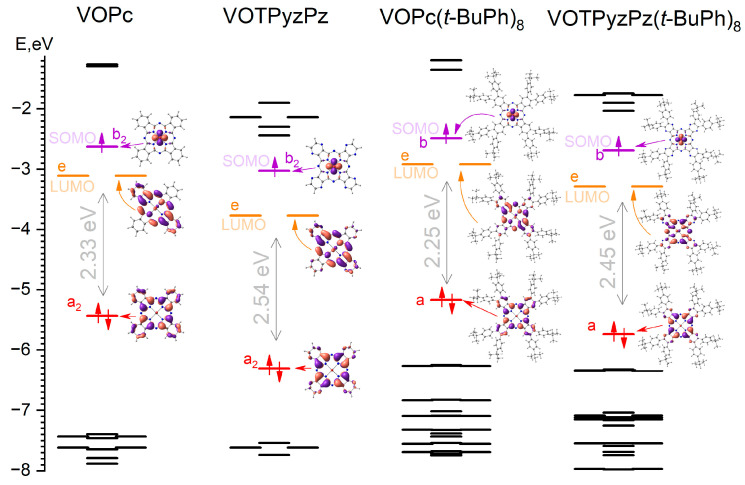
MO energy level diagram (ROPBE0-D3BJ/def2-TZVP/gas) and composition of frontier MOs for **VOPc**, **VOTPyzPz**, **VOPc(*t*-BuPh)_8_**, and **VOTPyzPz(*t*-BuPh)_8_**. MO level diagram for [**Pc**]^2−^ and [**TPyzPz**]^2−^ is shown in Figure S15.

As previously noted [48], the ground electronic state of **VOPc** is ^2^B_2_. As in the case of vanadyl octaethylporphyrin [49], the singly occupied molecular orbital (SOMO) is a d_xy_ orbital of the vanadium atom (Figure 5, as well as [48,50,51]). Unlike porphyrins, occupied Gouterman’s orbitals a_1u_ and a_2u_ (in D_4h_ terms) become more separated in **Pc**s [52] and **TPyzPz**s (Figure S15 [53]). The highest doubly occupied molecular orbital for **VOPc** is a_2_ (a_1u_ in D_4h_ terms) localized on the isoindole fragment (Figure 5). The lowest unoccupied molecular orbitals in **Pc**s and **TPyzPz**s are degenerate π* orbitals (Figure 5 and Figure S15). The addition of *t*-BuPh substituents to the macrocyclic skeletons, although it leads to a slight increase in the energy of the mentioned frontier orbitals, has little effect on the energy gap between Gouterman’s unoccupied and occupied orbitals e and a (e_g_ and a_1u_ in D_4h_ symmetry terms). The π-π* transition between these macrocyclic orbitals a_2_ and e determines the intense Q band ~650–730 nm. The energy gap between Gouterman’s unoccupied and occupied orbitals e and a (e_g_ and a_1u_ in D_4h_ symmetry terms) in **TPyzPz**s significantly decreases by 0.2 eV compared to those of **Pc**s (Figure 5), which determines the blue shift in the Q band in **VOTPyzPz(*t*-BuPh)_8_** compared to **VOPc(*t*-BuPh)_8_** (Figure S9).

**Figure 6:**
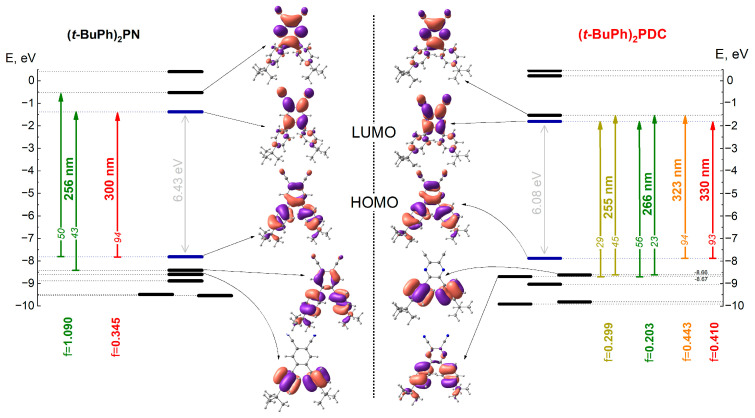
MO energy level diagram (CAM-B3LYP-D3BJ/def2-TZVP/PCM:DCM//PBE0-D3BJ/def2-TZVP/gas), composition of canonical Mos, and visual representation of several transitions for **(*t*-BuPh)_2_PN** (in **left**) and **(*t*-BuPh)_2_PDC** (in **right**). f denotes the oscillator strengths of the corresponding transitions. The composition of the excited states is shown in italics; transitions are listed if their contributions are greater than 10%. To improve the visual presentation of the diagram, the arrangement of accidentally degenerate HOMO-1 and HOMO-2 for **(*t*-BuPh)_2_PDC** has been changed; the correct values (−8.66 and −8.67 eV) of the energies of these orbitals are indicated. Arrows of different colors denote excitations to different excited states. Double gray arrows denote HOMO-LUMO gaps.

The HOMOs for **(*t*-BuPh)_2_PN**/**(*t*-BuPh)_2_PDC** are distributed throughout the entire π-conjugated molecules (mostly throughout the phenyl rings (~70%) and PN / PDC (~23%) moieties, Figure 6). The LUMOs are localized on the PN/PDC skeleton (~85%). For **(*t*-BuPh)_2_PDC**, the HOMO-LUMO energy gap is slightly narrower, by 0.35 eV than that for **(*t*-BuPh)_2_PN** (Figure 6). This results in a bathochromic shift in the observed band in the spectrum of **(*t*-BuPh)_2_PDC** in comparison with **(*t*-BuPh)_2_PN** (Figure S10). Atoms X_1_ and X_4_ make a significant contribution to the LUMO + 1, due to which these orbitals decrease their energy in the case of **(*t*-BuPh)_2_PDC** compared to **(*t*-BuPh)_2_PN**. Figure S10 shows satisfactory agreement between the theoretical and experimentally recorded absorption spectra.

### 2.6. IR Spectra

The position of bands in the simulated IR spectra of dinitriles are in satisfactory agreement with appropriate values from the experimental spectra measured in the solid phase (Figures S3 and S4) and with the literature [29,30]. The correlation between the bands of the simulated and experimental spectra is close to linear (scaling coefficients are in the range from 0.941 to 0.957, Figures S16 and S17). The IR spectra of the two dinitriles have a large number of bands in the range from 400 to 1650 cm^−1^, several bands in the range from 2800 to 3100 cm^−1^ corresponding to C-H vibrations, and one band at ~2230 cm^−1^ corresponding to C-N vibrations of the cyano-groups (Figures S3, S4, and S18). For **(*t*-BuPh)_2_PDC**, the band at ~2236 cm^−1^ is very weak in the spectra registered in the attenuated total reflectance mode, but this band is quite well manifested in the case of spectrum measured in KBr pellets (Figure S18). According to calculations, some C-H vibrations of butyl groups have large IR intensities and determine the bands at ~2900 cm^−1^ and 2980 cm^−1^ (Figures S3b and S4b). Weak bands with ω > 3020 cm^−1^ correspond to C-H vibrations in aromatic rings (Figures S3 and S4). Assignment of the other bands in the obtained IR spectra is presented in Tables S1 and S2.

For **VOPc(*t*-BuPh)_8_** and **VOTPyzPz(*t*-BuPh)_8_**, the band positions in the simulated IR spectra exhibit satisfactory agreement with the corresponding values obtained from the experimental spectra for the solid phase (Figures S6 and S8). In macroheterocyclic metal complexes (e.g., [54]), including the compounds under study, the observed IR bands typically arise from the superposition of some vibrational modes, making definitive assignment exceedingly difficult. In the region above 2800 cm^−1^, the spectra of vanadyl complexes resembles those of the precursor dinitriles, as the bands in this region correspond to C-H vibrations. The band at ν_exp_ = 1094 cm^−1^ is the most intense band in the spectrum of **VOPc(*t*-BuPh)_8_**. It may correspond to a vibration involving deformation of the isoindole fragments with a large contribution from stretching of the C-N and C-C bonds (ω_th_ = 1105 cm^−1^, Figure S6b). For **VOTPyzPz(*t*-BuPh)_8_**, the same vibration corresponds to a band ω_th_ = 1128 cm^−1^ (Figure S8b). Stretching of the V=O bond (ω_th_= 1094 and 1098 cm^−1^ in the two cases) has a rather high IR intensity, but its closeness to bands ω_th_ = 1105 and 1128 cm^−1^, which have even greater intensities, can make it difficult to isolate in the spectra (Figures S6 and S8). According to literature data, the calculated value of V=O vibration in porphyrin complexes is ∼1100 cm^−1^ [55], while it is manifests in the experimental IR spectra in the region of ∼1000 cm^−1^ [56,57,58]. For **VOPc(*t*-BuPh)_8_** and **VOTPyzPz(*t*-BuPh)_8_**, the high-intensity vibrations with ω_th_ = 1329 and 1345 cm^−1^ are mostly attributed to the N-C and C-C stretching in the isoindole moieties and apparently appear at ν_exp_ = 1325 and 1346 cm^−1^ in the experimental spectra (Figures S6 and S8). The bands at 1608 and 1607 cm^−1^ in the theoretical spectra correspond to C-C stretching in the phenyl moieties.

The bands at ν_exp_= 833 and 839 cm^−1^ in the experimental spectra of **VOPc(*t*-BuPh)_8_** and **VOTPyzPz(*t*-BuPh)_8_** are assigned to the C-H out-of-plane bending (ω_th_ = 819 and 823 cm^−1^, Figures S6 and S8). The bands at ν_exp_= 731 and 727 cm^−1^ are assigned to a degenerate vibrational mode related to the complicated deformation of the entire molecule (ω_th_ = 715 and 714 cm^−1^, Figures S6 and S8). The bands at ν_exp_= 939 and 949 cm^−1^ are attributed to another degenerate vibrational mode, also associated with complex deformation of the entire molecule (ω_th_ = 920 and 933 cm^−1^, Figures S6 and S8).

## 3. Materials and Methods

### 3.1. Synthesis

**(*t*-BuPh)_2_PN** *(4,5-bis(4-tert-butylphenyl)phthalonitrile).* A mixture of 4,5-dichlorophthalonitrile (2.00 g, 0.01 mmol), 4-*tert*-butylphenylboronic acid (7.12 g, 0.04 mmol), KBr (1.2 g, 0.01 mmol), and a saturated aqueous solution of K_2_CO_3_ (5.6 g, 0.04 mmol) was stirred in 70 mL of boiling 1,4-dioxane under argon. After the solvent began to boil, the dichloro-bis(triphenylphosphine) palladium compound (0.12 g, 0.0002 mmol) was added. The reaction was carried out for 6 h. The reaction mixture was cooled to room temperature and water was added. The product was collected by extraction with ethyl acetate. After rotary evaporation, greenish-gray powder was purified by flash chromatography on silica gel (ethyl acetate/*n*-hexane (1:10)). The obtained product was additionally purified by sublimation at 250 °C to give a white powder (2.9 g, 74%). ^1^H NMR (500 MHz, CDCl_3_): δ 7.84 (s, 2H), 7.31 (d, J = 8.53 Hz, 4H), 7.05 (d, J = 8.53 Hz, 4H), 1.32 (s, 18H) (Figure S1). ^13^C NMR (500 MHz, CDCl_3_): δ 151.8, 145.8, 135.6, 134.8, 129.3, 125.7, 115.5, 114, 34.6, 31.2 (Figure S2). IR: 573, 594, 833, 928, 1015, 1113, 1269, 1364, 1462, 1483, 1589, 1609, 2237, 2961 cm^−1^ (Figure S3).

**(*t*-BuPh)_2_PDC** *(5,6-bis(4-tert-butylphenyl)pyrazine-2,3-dicarbonitrile; 5,6-bis(4-tert-butylphenyl)-2,3-dicyanopyrazin; 2,3-dicyano-5,6-bis(4-tertbutylphenyl)pyrazine)* was obtained by slight modification of a known method [28]. A mixture of 9.6 g (30 mmol) of 4,4′-bis(*tert*-butylphenyl)-ethane-1,2-dione and 3.24 g (30 mmol) of diaminomaleonitrile was heated in 60 mL of glacial acetic acid for 2.5 h under reflux. After completion of the reaction, acetic acid was evaporated by a rotary evaporator, and the crude product was redissolved in dichloromethane (DCM) and purified by flash chromatography on silica gel using DCM as the eluent. As a result, a pale yellow powder of the desired product was obtained in 88% yield. IR: 577, 600, 845, 939, 1015, 1117, 1198, 1227, 1375, 1466, 1504, 1601, 2236, 2965 cm^−1^ (Figure S4).

**VOPc(*t*-BuPh)_8_** *(vanadyl octa-(4-tert-butylphenyl)phthalocyanine).* A mixture of 39 mg (0.1 mmol) of **(*t*-BuPh)_2_PN** and 4 mg of VCl_3_ (0.025 mmol) was dissolved in 1 mL of 1,2,4-thrichlorobenzene and refluxed for 2 h. After cooling to room temperature, the reaction mixture was poured into ethanol and centrifuged. The greenish powder was washed three times with ethanol and finally purified by column chromatography on silica gel using DCM as the eluent to give a grass-green powder (11 mg, 27%). UV-Vis (DCM): λmax (A/Amax) = 722 (1), 648 (0.18), 428 (0.07). MALDI-TOF (DHB, positive): 1638.5 [M + H^+^] (calcd. for C_112_H_112_N_8_OV 1637.1) (Figure S5). IR: 731, 758, 833, 939, 1009, 1094, 1269, 1298, 1325, 1395, 1404, 1443, 1611, 2961 cm^−1^ (Figure S6).

**VOTPyzPz(*t*-BuPh)_8_** *(vanadyl octa-(4-tert-butylphenyl)tetrapyrazinoporphyrazine)*. A mixture of 200 mg (0.5 mmol) of **(*t*-BuPh)_2_PDC** and 20mg (0.125 mmol) of VCl_3_ was melted at 220 °C for 15 min. After cooling, the dark powder was washed with ethanol and finally purified by column chromatography on silica gel using DCM as the eluent to give a dark-green powder (150 mg, 72%). UV-Vis (DCM): λmax (A/Amax) = 659 (1), 599 (0.14), 459 (0.07), 369 (0.44). MALDI-TOF (DHB, positive): 1646.70 [M + H^+^] (calcd. for C_104_H_104_N_16_OV 1645.03) (Figure S7). IR: 685, 727, 797, 839, 949, 1065, 1099, 1117, 1238, 1248, 1346, 1462, 1531, 1609, 2955 (Figure S8).

All other chemicals for the syntheses were purchased from certified suppliers (i.e., Sigma-Aldrich (St. Louis, MO, USA), TCI (Tokyo, Japan), EKOS-1 (Moscow, Russia)) and used as received.

### 3.2. Spectral Study

UV-V is spectra were recorded using a Jasco V-770 spectrophotometer (Jasco, Tokyo, Japan) in DCM. The IR spectra were measured on a Shimadzu IRAffinity-1 (Shimadzu, Kyoto, Japan) Fourier transform IR spectrophotometer equipped with a Specac Quest ATR Diamond GS10800-B (Specac, London, UK) accessory in the mid-infrared region (400–4000 cm^−1^). Mass spectra were recorded on an MALDI TOF Shimadzu Biotech Axima Confidence (Shimadzu, Kyoto, Japan) mass spectrometer using the resources of the Center for Shared Use of Scientific Equipment of the ISUCT (Ivanovo, Russia).

### 3.3. Electrochemical Study

Cyclic voltammetry (CVA) study was performed in a three-electrode electrochemical cell with a glassy carbon working electrode, a Pt wire counter electrode, and a Ag/AgCl reference electrode on a Smart Stat potentiostat–galvanostat PS-50, in argon-deoxygenated DCM with 0.1 M tetrabutylammonium perchlorate as the supporting electrolyte; the reference ferrocene/ferrocenium (Fc/Fc^+^) couple was observed at +0.507 V. Ferrocene was used as an internal standard by adding it to the solution containing the compound being studied.

### 3.4. Computational Details

Quantum chemical calculations for the dinitriles and the corresponding vanadyl complexes were carried out using the Gaussian09 program [59]. The calculations were performed using the PBE0 functional [60,61] and the def2-TZVP [62] basis set, with D3 version of the Grimme’s dispersion with Becke–Johnson damping (D3BJ) [63], “Tight” optimization convergence criteria (maximum force = 1.5∙10^−5^ H·Bohr^–1^, root mean square (RMS) force = 1.0∙10^−5^ H·Bohr^–1^, maximum displacement = 6.0∙10^−5^ Å, RMS displacement = 4.0∙10^−5^ Å), “ultrafine” grid (pruned, 99 radial shells and 590 angular points per shell). Basis sets and ECPs were taken from the Basis Set Exchange library [64]. In order to obtain theoretical IR spectra for **VOPc(*t*-BuPh)_8_** and **VOTPyzPz(*t*-BuPh)_8_**, PBE0-D3BJ/def2-SVP [62] calculations were used. The results of harmonic frequencies calculations with different basis sets were compared using **(*t*-BuPh)_2_PN** and **(*t*-BuPh)_2_PDC** as examples (Figures S3, S4, S16, and S17). The assignments of vibrational modes of **(*t*-BuPh)_2_PN** and **(*t*-BuPh)_2_PDC** were carried out based on potential energy distribution (PED) analysis of PBE0-GD3BJ/def2-TZVP results among internal coordinates using the VibModule program (version 1.3.2) [65].

Simulations of electronic absorption spectra were performed by time-dependent density functional theory (TDDFT) calculations with CAM-B3LYP [66], D3BJ, and the def2-TZVP basis set for PBE0-D3BJ/def2-TZVP geometries. The simplified TDDFT (sTDDFT) [67] approach, as implemented in the ORCA 5.0.3 software [68], was also used to simulate UV-Vis spectra. Visualization of the molecular structures and orbitals was performed using the ChemCraft program (version 1.8) [69]. The contributions of atomic orbitals of specific atoms to molecular orbitals were calculated based on the results of QC calculations using the GausSum program (version 3.0.2) [70].

The bands in the simulated IR and UV-Vis spectra (Figures S3, S4, S6, S8–S10 and S12) were described by Lorentz curves with a full width at half maximum of 10 cm^−1^ and 10 nm, respectively.

## 4. Conclusions

The existing synthesis method of 4,5-bis(4-*tert*-butylphenyl)phthalonitrile (**(*t*-BuPh)_2_PN**) and 5,6-bis(4-*tert*-butylphenyl)pyrazine-2,3-dicarbonitrile (**(*t*-BuPh)_2_PDC**) were modified at the purification stage. Two vanadyl complexes, **VOPc(*t*-BuPh)_8_** and **VOTPyzPz(*t*-BuPh)_8_**, were obtained by tetramerization of these nitriles with VCl_3_. The basic properties were studied in TFA and sulfuric acid. An absorption spectrum recorded in pure sulfuric acid coincides with that of the diprotonated form of **VOPc(*t*-BuPh)_8_**. In the case of **VOTPyzPz(*t*-BuPh)_8_**, protonation of the pyrazine rings was observed. In the CVAs, the first reduction potential shifted by approximately 0.4 V toward zero when benzene rings were replaced by pyrazine rings in both the dinitrile and vanadyl complexes due to LUMO stabilization. Comparison of our results with data for related structures revealed that the primary role is played not by the peripheral substituents, but by the ring annelated to the porphyrazine hull. The phenyl groups are rotated relative to the molecule backbone and are located in a “quasi-parallel manner”. A bathochromic shift in the longest wavelength band in the electronic absorption spectrum of **(*t*-BuPh)_2_PDC** was observed in comparison with **(*t*-BuPh)_2_PN** due to a narrower HOMO-LUMO gap for the former. It was shown that **VOPc(*t*-BuPh)_8_** and **VOTPyzPz(*t*-BuPh)_8_** have dome-shaped distortions and can be characterized by conformational multiformity caused by the possibility of different orientations of the four pairs of phenyl groups, as well as *tert*-butyl group rotation. It was noted that the addition of *t*-BuPh substituents to the macrocyclic skeleton of **VOPc** or **VOTPyzPz** has little effect on the energy gap between Gouterman’s unoccupied and occupied orbitals and, consequently, on the Q band position. Two complexes, **VOPc(*t*-BuPh)_8_** and **VOTPyzPz(*t*-BuPh)_8_**, were synthesized for the first time. The syntheses of their precursors, **(*t*-BuPh)_2_PN** and **(*t*-BuPh)_2_PDC**, were modified, resulting in higher yields. The structural, spectral, and electrochemical properties of the four compounds were thoroughly studied using a complex approach that included both theoretical and experimental methods. The results obtained will be useful for the further development of the chemistry of vanadyl macroheterocyclic compounds.

## Figures and Tables

**Table 1:** Maxima of the Q band for meso-protonated forms and their bathochromic shifts. Q_0_ is the value of the Q band in a neutral molecule and Q_n_ is the value of the band maximum at the *n*-th protonation stage.

Compound	λabs (Q_0_–Q_n_), nm (cm^−1^)				
	*n* = 0	*n* = 1	*n* = 2	*n* = 3	*n* = 4
[Al(Cl)PcCl_8_] *	681	710 (600)	734 (1060)	774 (1764)	810 (2339)
[Ga(OH)PcCl_8_] *	682	-	728 (926)	774 (1740)	811 (2330)
[In(OH)PcCl_8_] *	688	-	-	-	818 (2289)
**[Al(OH)Pc(*t*-Bu)_4_] #**	691	718 (540)	740 (949)	805 (2044)	825 (2344)
**[Ga(OH)Pc(*t*-Bu)_4_] #**	693	718 (510)	744 (980)	802 (1970)	835 (2410)
**[In(OH)Pc(*t*-Bu)_4_] #**	695	727 (650)	745 (960)	799 (1870)	830 (2320)
**[VOPc(*t*-BuPh)_8_]**	722	777(980)	801(1366)	-	-
**[VOTPyzPz(*t*-BuPh)_8_]**	662	687(550)	-	-	-

#—data from ref. [32]; *—data from ref. [33].

**Table 2:** Reduction potentials of studied and related compounds.

Compound	Reduction Potentials *E*_1/2_, V			Solvent|Reference Electrode	References
	1st	2nd	3rd		
**(*t*-BuPh)_2_PN**	−1.57	-	-	DCM|Ag/AgCl	This Work
***(t*** **-BuPh)_2_PDC**	−1.13	-	-	DCM|Ag/AgCl	This Work
**VOPc(*t*-BuPh)_8_**	−0.58	−0.90	-	DCM|Ag/AgCl	This Work
**VOTPyzPz(*t*-BuPh)_8_**	−0.19	−0.49	−0.77	DCM|Ag/AgCl	This Work
**VOTPyzPzCl_8_**	−0.19	−0.52	−0.83	DMF|Ag/AgCl	[18]
**VOPc(NaphtO)_4_**	−0.64	−0.98	−1.83	DCM|SCE	[36]
**VOPc(*t*-Bu)_2_C_6_H_3_O)_4_**	−0.51	−0.97	−1.94	DMF|SCE	[37]
**VOPc(C_8_H_17_O)_4_**	−0.62	−1.12	−2.07	DMF|SCE	[37]
**VOPc**	−0.58	−1.08	-	DMF	[38]
**VOTBP**	−1.19	−1.42	-	DMF|Ag/AgCl	[39]
**VOTTDPz**	−0.4	-	-	Thin film|Ag/AgCl	[40]

**Table 3:** Estimated values of frontier MO levels.

Compound	*E_LUMO_* ^a^, eV		*OE_G_* or Δ(HOMO-LUMO), eV		*E_HOMO_* ^b^, eV	
	CVA ^c^	PBE0 ^d^	UV-Vis ^e^	PBE0 ^d^	Exp ^f^	PBE0 ^d^
**(*t*-BuPh)_2_PN**	−2.31	−2.36	3.49	4.52	−5.80	−6.88
**(*t*-BuPh)_2_PDC**	−2.83	−2.76	3.10	4.18	−5.93	−6.94
**VOPc(*t*-BuPh)_8_**	−3.49	−2.92	1.66	2.25	−5.15	−5.17
**VOTPyzPz(*t*-BuPh)_8_**	−3.95	−3.29	1.81	2.45	−5.76	−5.74

^a^ the shapes of LUMOs are shown below; ^b^ for **VOPc(*t*-BuPh)_8_** and **VOTPyzPz(*t*-BuPh)_8_**—doubly occupied MO of symmetry a; ^c^ calculated by Equation (1) using experimental CVA data; ^d^ according to ROPBE0-D3BJ/def2-TZVP/gas calculations; ^e^ calculated by Equation (2) using experimental UV-Vis data; ^f^ calculated by Equation (3) using experimental UV-Vis and CVA data.

**Table 4:** Selected structural parameters ^a^ of dinitriles by PBE0-D3BJ/def2-TZVP/gas calculations.

Parameters ^a^	PN	Ph_2_PN	(*t*-BuPh)_2_PN	PDC	Ph_2_PDC	(*t*-BuPh)_2_PDC
Symmetry	C_2v_	C_2_	C_2_	C_2v_	C_2_	C_2_
C_5_-C_6_	1.388	1.407	1.408	1.393	1.423	1.425
X_1_-C_6_	1.384	1.391	1.391	1.321	1.326	1.326
X_1_-C_2_	1.392	1.388	1.389	1.329	1.325	1.326
C_2_-C_3_	1.404	1.402	1.402	1.403	1.398	1.398
C_2_-C^CN^	1.425	1.424	1.424	1.432	1.431	1.431
C^CN^-N^CN^	1.151	1.151	1.151	1.150	1.150	1.150
C_6_-C_1_^Ph^	-	1.478	1.476	-	1.472	1.470
C_1_^Ph^-C_2_^Ph^	-	1.394	1.395	-	1.394	1.395
C_3_^Ph^-C_4_^Ph^	-	1.388	1.397	-	1.388	1.397
C_4_^Ph^-C_1_^Bu^	-	-	1.522	-	-	1.522
φ_1_ = φ_2_ ^b^	-	50.1	49.5	-	38.1	37.3

^a^ internuclear distances r_e_—in Å, valence angles—in °; designations of atoms and angles are shown in Figure 3. ^b^ rotation angles of the two phenyl rings (see in Figure 3).

## Data Availability

The original contributions presented in this study are included in the article/Supplementary Material. Further inquiries can be directed to the corresponding authors.

## Supplementary Materials

The following supporting information can be downloaded at: https://www.mdpi.com/article/10.3390/ijms27020606/s1.
