# Supplementary material for: Influence of Aza-Substitution on Molecular Structure, Spectral and Electronic Properties of t-Butylphenyl Substituted Vanadyl Complexes
Source: Int J Mol Sci. 2026 Jan 7;27(2):606. doi: 10.3390/ijms27020606 (PMC12840634; doi:10.3390/ijms27020606)
Supplement: Supplementary file 1 [file ijms-27-00606-s001.zip › ijms-4068000-supplementary.pdf]

Supporting information for

Influence of aza-substitution on molecular structure, spectral and electronic properties of *t*-butylphenyl substituted vanadyl complexes

Daniil N. Finogenov*, Alexander E. Pogonin, Yuriy A. Zhabanov, Ksenia V. Ksenofontova, Dominika Yu. Parfyonova, Alexey V. Eroshin and Pavel A. Stuzhin

Research Institute of Chemistry of Macroheterocyclic Compounds, Ivanovo State University of Chemistry and Technology, Sheremetevskiy av. 7, 153000 Ivanovo, Russia

Contents

Figure S1. ^{1}H NMR spectrum of **(*t*-BuPh)$_2$PN**...3

Figure S2. ^{13}C NMR spectrum of **(*t*-BuPh)$_2$PN**...3

Figure S3. IR spectra for **(*t*-BuPh)$_2$PN**: (a) experimental spectrum, (b) simulated (scaled with coefficient = 0.957, Figure S6) spectrum based on PBE0-D3BJ/def2-TZVP calculations, (c) simulated (scaled with coefficient = 0.951, Figure S6) spectrum based on PBE0-D3BJ/def2-SVP calculations, (d) unscaled theoretical (PBE0-D3BJ/def2-TZVP) spectrum, (e) unscaled theoretical (PBE0-D3BJ/def2-SVP) spectrum. In order to simulate the experimental shape of IR spectrum, for the results of QC calculations the individual bands were described by Lorentz curves with a full width at half maximum of 10 cm^{-1}. 4

Figure S4. IR spectra for **(*t*-BuPh)$_2$PDC**: (a) experimental spectrum, (b) simulated (scaled with coefficient = 0.950, Figure S17) spectrum based on PBE0-D3BJ/def2-TZVP calculations, (c) simulated (scaled with coefficient = 0.941, Figure S17) spectrum based on PBE0-D3BJ/def2-SVP calculations, (d) unscaled theoretical (PBE0-D3BJ/def2-TZVP) spectrum, (e) unscaled theoretical (PBE0-D3BJ/def2-SVP) spectrum. In order to simulate the experimental shape of IR spectrum, for the results of QC calculations the individual bands were described by Lorentz curves with a full width at half maximum of 10 cm^{-1}. 5

Figure S5. MALDI TOF mass spectrum of **VOPc(*t*-BuPh)$_8$**..6

Figure S6. IR spectra for **VOPc(*t*-BuPh)$_8$**: (a) experimental spectrum, (b) simulated (scaled with coefficient = 0.946) spectrum based on PBE0-D3BJ/def2-SVP calculations, (c) unscaled theoretical (PBE0-D3BJ/def2-SVP) spectrum. In order to simulate the experimental shape of IR spectrum, for the results of QC calculations the individual bands were described by Lorentz curves with a full width at half maximum of 10 cm^{-1}. 6

Figure S7. MALDI TOF mass spectrum of **VOTPyzPz(*t*-BuPh)$_8$**..7

Figure S8. IR spectra for **VOTPyzPz(*t*-BuPh)$_8$**: (a) experimental spectrum, (b) simulated (scaled with coefficient = 0.946) spectrum based on PBE0-D3BJ/def2-SVP calculations, (c) unscaled theoretical (PBE0-D3BJ/def2-SVP) spectrum. In order to simulate the experimental shape of IR spectrum, for the results of QC calculations the individual bands were described by Lorentz curves with a full width at half maximum of 10 cm^{-1}. 7

Figure S9. Experimental UV-Vis spectra for **VOPc(*t*-BuPh)$_8$** (a) and **VOTPyzPz(*t*-BuPh)$_8$** (c) in DCM in comparison with simulated (sTDDFT/CAM-B3LYP-D3BJ/def2-TZVP/CPCM:DCM // PBE0-D3BJ/def2-TZVP/gas) electronic absorption spectra for **VOPc(*t*-BuPh)$_8$** (b) and **VOTPyzPz(*t*-BuPh)$_8$** (d). 8

Figure S10. Experimental UV-Vis spectra for **(*t*-BuPh)$_2$PN** (a) and **(*t*-BuPh)$_2$PDC** (c) in DCM in comparison with simulated (CAM-B3LYP-D3BJ/def2-TZVP/CPCM:DCM // PBE0-D3BJ/def2-TZVP/gas) electronic absorption spectra for **(*t*-BuPh)$_2$PN** (b) and **(*t*-BuPh)$_2$PDC** (d). 9

Figure S11. Relaxed potential energy function (PBE0-D3BJ/def2-SVP/gas) of internal symmetrical (φ_1=φ_2) rotation of two *t*-BuPh-groups in **(*t*-BuPh)$_2$PN** and **(*t*-BuPh)$_2$PDC** molecules. The calculations were carried out with two frozen dihedral angles φ_1=φ_2. For structures with φ_1=φ_2< φ_{opt}, there is a significant deviation of χ(C$_5$-X$_1$-C$_6$-C$_1^{Ph}$) from 180 ° due to the substantial steric repulsions caused by the close proximity of the phenylene rings...............10

Figure S12. Normalized calculated TDDFT (CAM-B3LYP-D3BJ/def2-TZVP/CPCM:DCM // PBE0-D3BJ/def2-TZVP/gas) electronic absorption spectra for ***t*-BuPh-PN** and ***t*-BuPh-PDC** with different rotation angles φ. Structures of ***t*-BuPh-PN** and ***t*-BuPh-PDC** with φ=35.5° and 16.7° correspond to minima on the potential energy surfaces...11

Figure S13. Relaxed potential energy function (PBE0-D3BJ/def2-TZVP/gas) of internal rotation of *t*-BuPh-group in ***t*-BuPhPN** and ***t*-BuPhPDC** molecules. 12

Figure S14. Conformer models of **VOPc(*t*-BuPh)$_8$**: left – C$_4$-structure; right – C$_{2v}$-structure. The color shows the spatial arrangement of atoms relative to the planes of isoindole moieties: red – above the planes, green – under the planes. 13

Figure S15. MO level diagram (ROPBE0-D3BJ/def2-TZVP/gas) and composition of frontier MOs for anions [porphyrin]$^{2-}$ - [**P**]$^{2-}$, [porphyrazine]$^{2-}$ - [**Pz**]$^{2-}$, [**Pc**]$^{2-}$ and [**TPyzPz**]$^{2-}$. 13

Figure S16. Correlation dependences ν_{exp} = f(ω_{th}): ν_{exp} and ω_{th} - the positions of the band maxima in the experimental and simulated (black squares ■ – PBE0-GD3BJ/def2-TZVP, Figure S3; red circles ● – PBE0-GD3BJ/def2-SVP, Figure S3) spectra for **(*t*-BuPh)$_2$PN**, respectively. R – adjusted R squared...14

Figure S17. Correlation dependences ν_{exp} = f(ω_{th}): ν_{exp} and ω_{th} - the positions of the band maxima in the experimental and simulated (black squares ■ – PBE0-GD3BJ/def2-TZVP, Figure S4; red circles ● – PBE0-GD3BJ/def2-SVP, Figure S4) spectra for **(*t*-BuPh)$_2$PDC**, respectively. R – adjusted R squared...15

Figure S18. Experimental IR spectra for **VOPc(*t*-BuPh)$_8$**: (a) in attenuated total reflectance mode; (b) in KBr pellets...15

Table S1. Calculated frequencies (ω_i), IR-intensities ($I_{IR\ i}$) and vibrational modes descriptions [a] for **(*t*-BuPh)$_2$PN.** 16

Table S2. Calculated frequencies (ω_i), IR-intensities ($I_{IR\ i}$) and vibrational modes descriptions [a] for **(*t*-BuPh)$_2$PDC.** 17

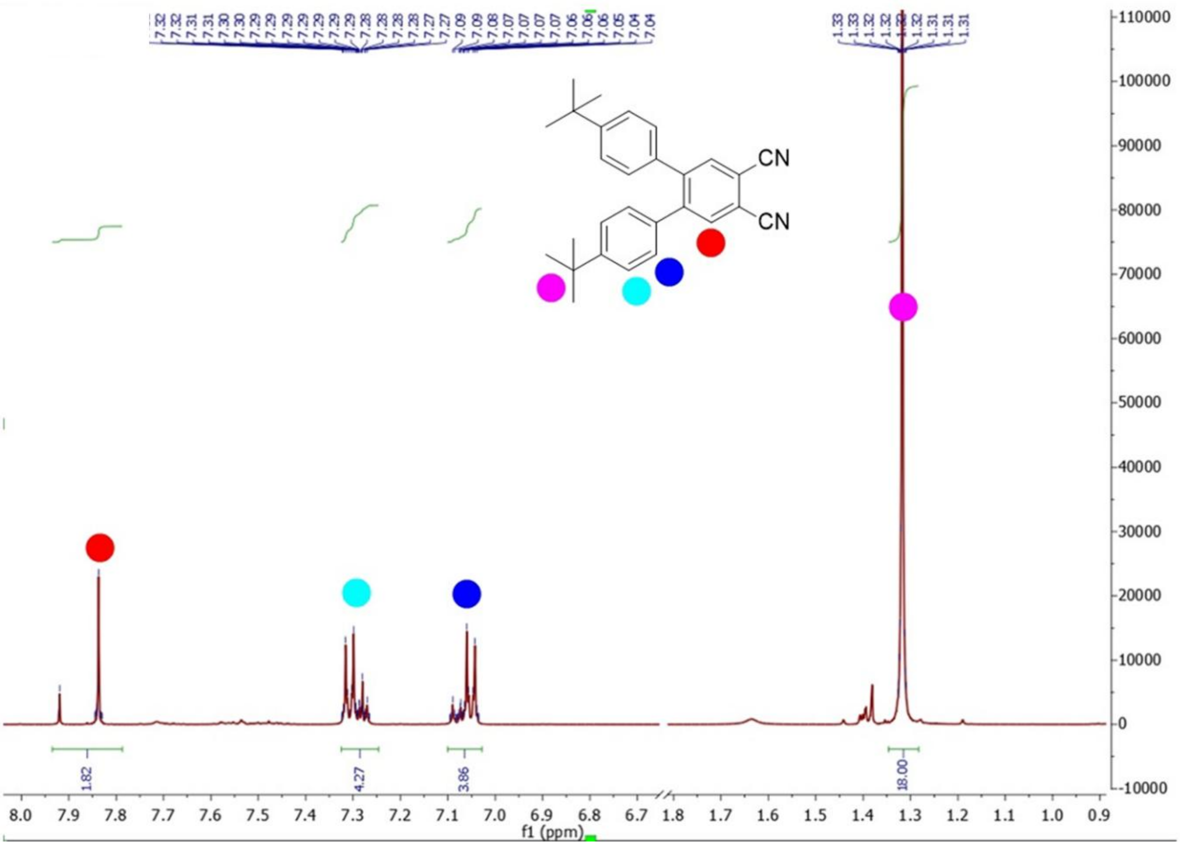

Figure S1. ^{1}H NMR spectrum of **(*t*-BuPh)$_2$PN**.

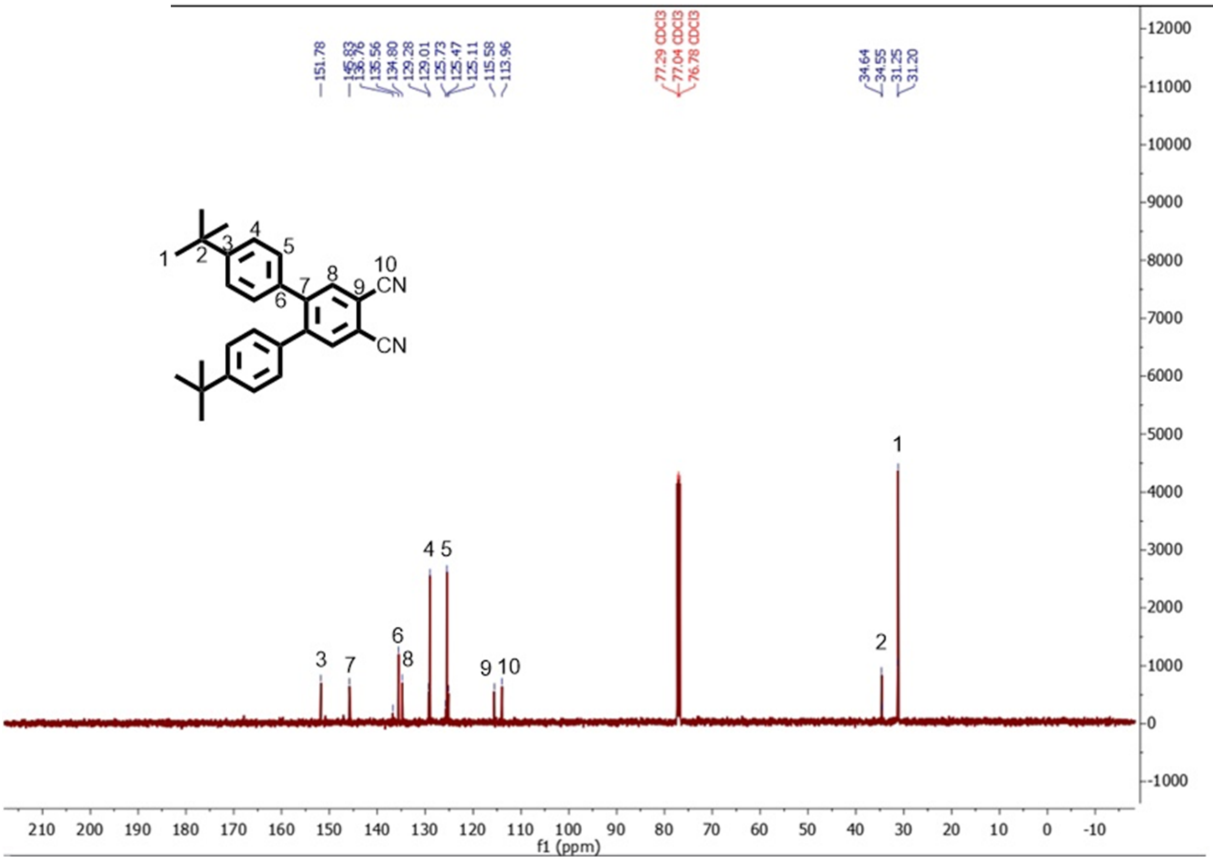

Figure S2. ^{13}C NMR spectrum of **(*t*-BuPh)$_2$PN**.

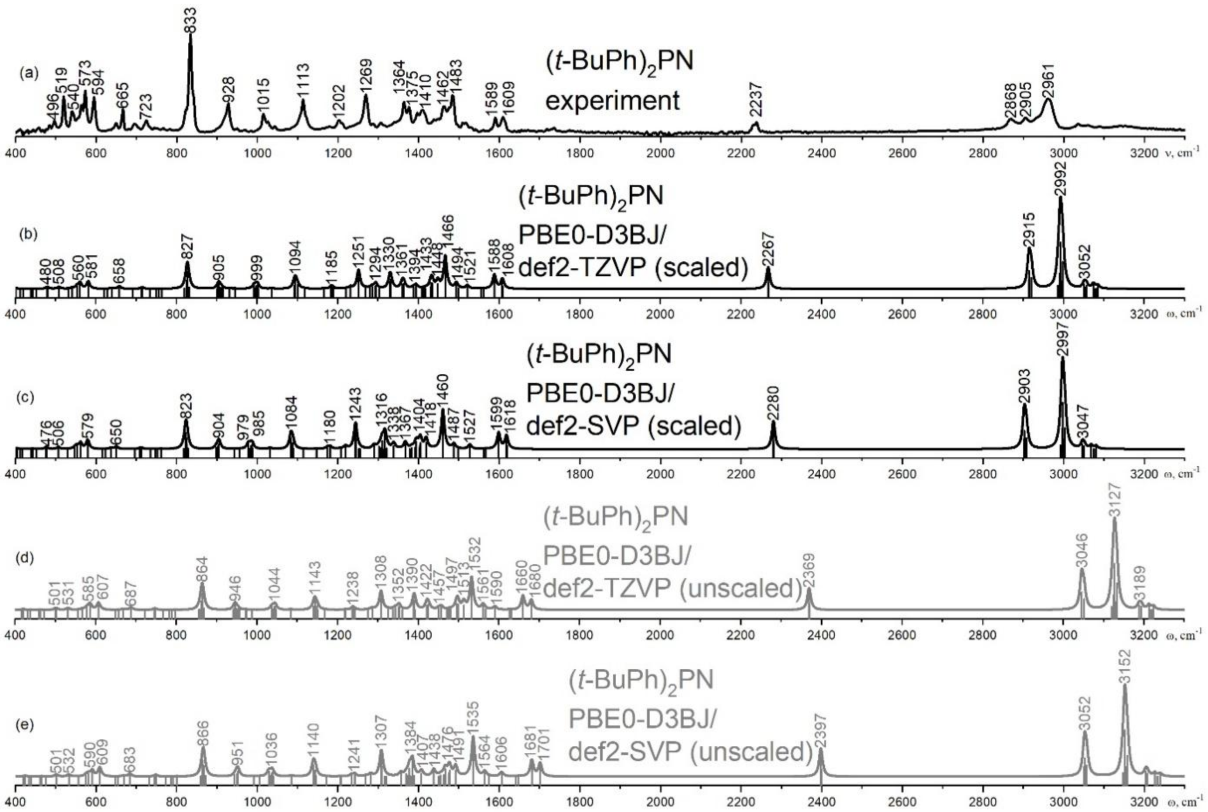

Figure S3. IR spectra for **(t-BuPh)$_2$PN**: (a) experimental spectrum, (b) simulated (scaled with coefficient = 0.957, Figure S6) spectrum based on PBE0-D3BJ/def2-TZVP calculations, (c) simulated (scaled with coefficient = 0.951, Figure S6) spectrum based on PBE0-D3BJ/def2-SVP calculations, (d) unscaled theoretical (PBE0-D3BJ/def2-TZVP) spectrum, (e) unscaled theoretical (PBE0-D3BJ/def2-SVP) spectrum. In order to simulate the experimental shape of IR spectrum, for the results of QC calculations the individual bands were described by Lorentz curves with a full width at half maximum of 10 cm^{-1}.

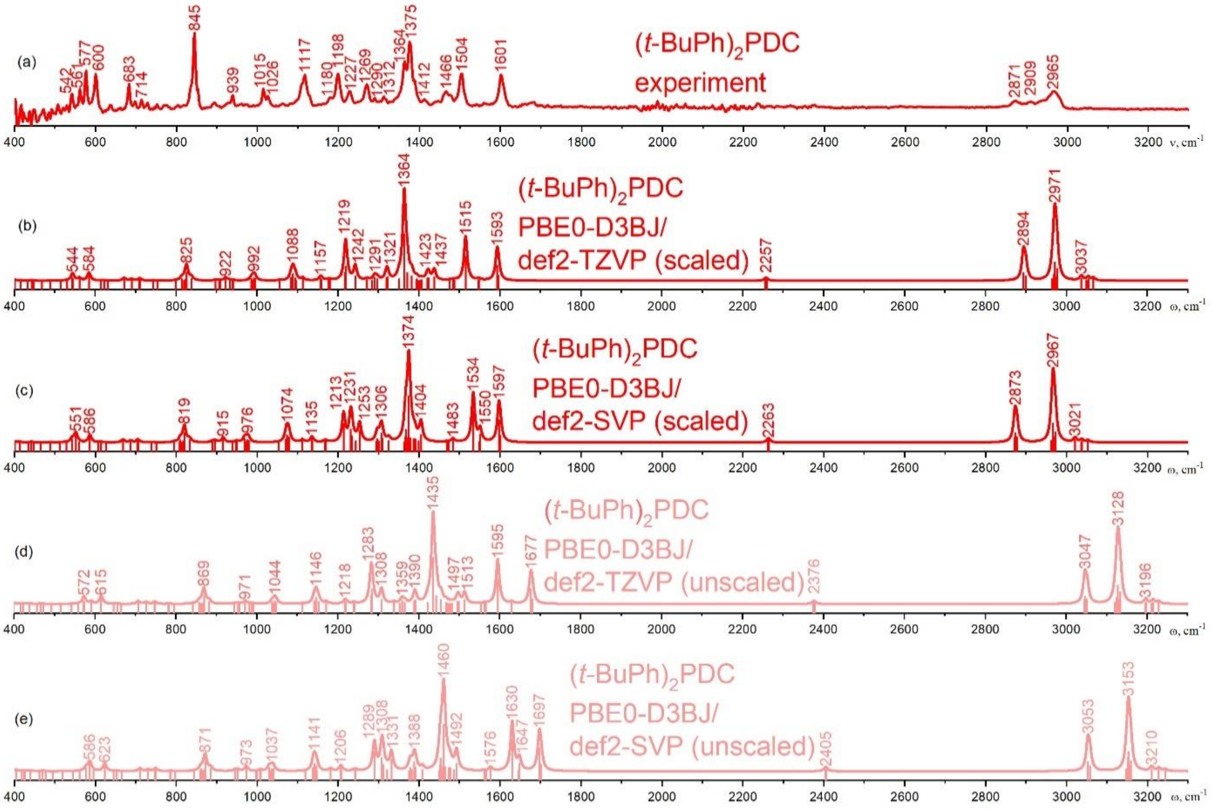

Figure S4. IR spectra for **(*t*-BuPh)$_2$PDC**: (a) experimental spectrum, (b) simulated (scaled with coefficient = 0.950, Figure S17) spectrum based on PBE0-D3BJ/def2-TZVP calculations, (c) simulated (scaled with coefficient = 0.941, Figure S17) spectrum based on PBE0-D3BJ/def2-SVP calculations, (d) unscaled theoretical (PBE0-D3BJ/def2-TZVP) spectrum, (e) unscaled theoretical (PBE0-D3BJ/def2-SVP) spectrum. In order to simulate the experimental shape of IR spectrum, for the results of QC calculations the individual bands were described by Lorentz curves with a full width at half maximum of 10 cm^{-1}.

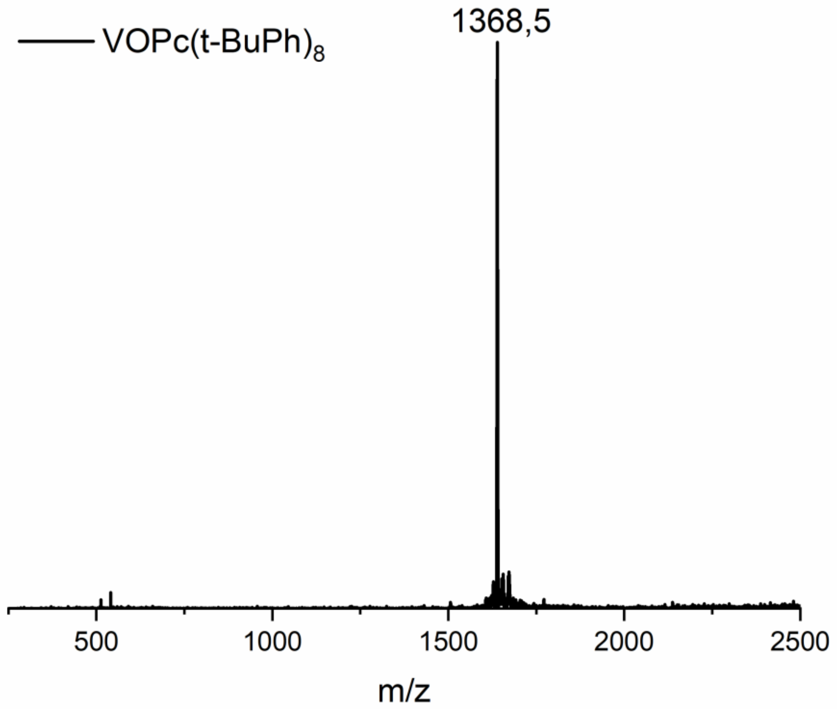

Figure S5. MALDI TOF mass spectrum of **VOPc(*t*-BuPh)$_8$**.

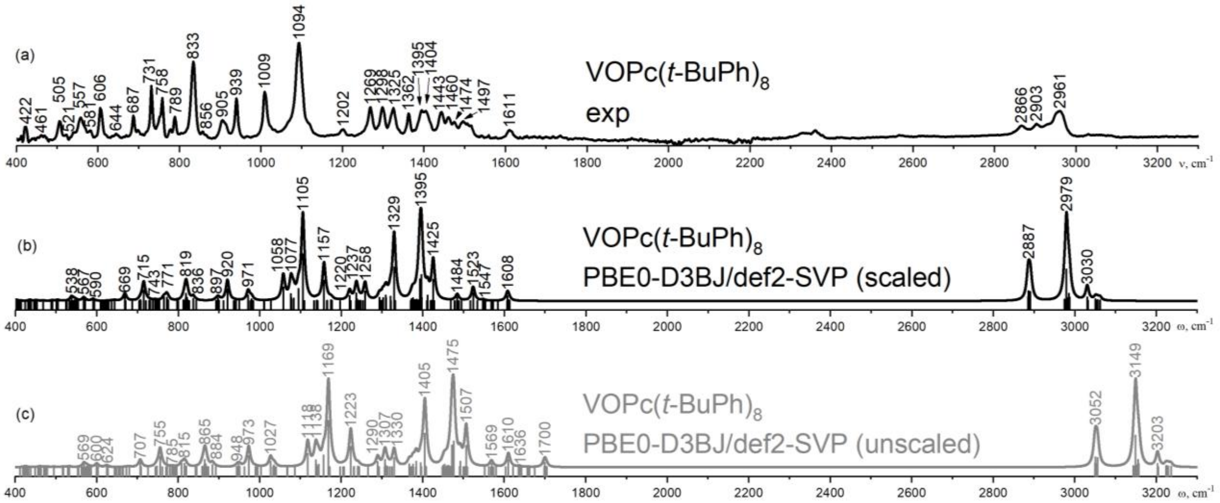

Figure S6. IR spectra for **VOPc(*t*-BuPh)$_8$**: (a) experimental spectrum, (b) simulated (scaled with coefficient = 0.946) spectrum based on PBE0-D3BJ/def2-SVP calculations, (c) unscaled theoretical (PBE0-D3BJ/def2-SVP) spectrum. In order to simulate the experimental shape of IR spectrum, for the results of QC calculations the individual bands were described by Lorentz curves with a full width at half maximum of 10 cm^{-1}.

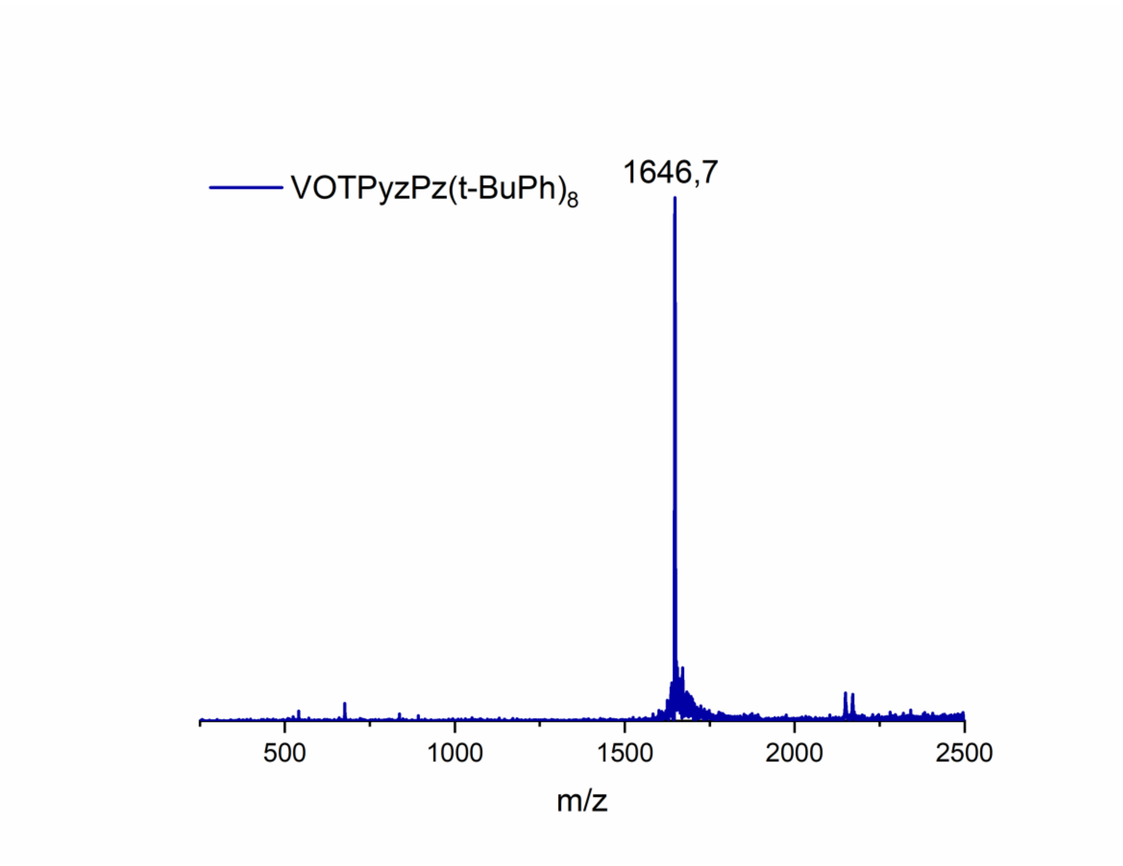

Figure S7. MALDI TOF mass spectrum of **VOTPyzPz(*t*-BuPh)$_8$**.

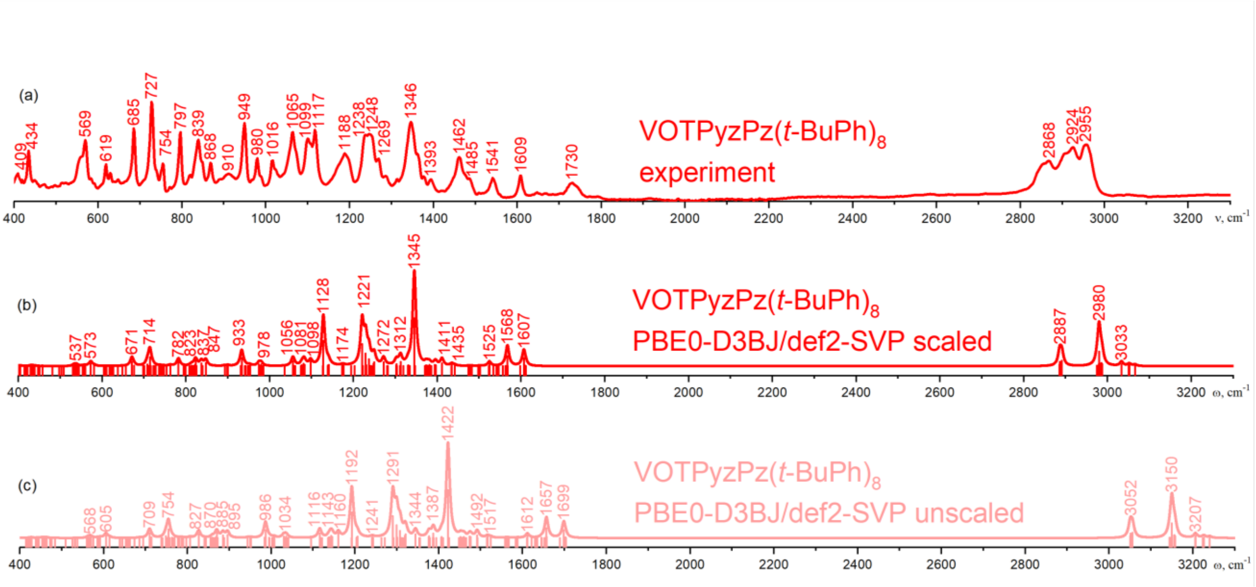

Figure S8. IR spectra for **VOTPyzPz(*t*-BuPh)$_8$**: (a) experimental spectrum, (b) simulated (scaled with coefficient = 0.946) spectrum based on PBE0-D3BJ/def2-SVP calculations, (c) unscaled theoretical (PBE0-D3BJ/def2-SVP) spectrum. In order to simulate the experimental shape of IR spectrum, for the results of QC calculations the individual bands were described by Lorentz curves with a full width at half maximum of 10 cm^{-1}.

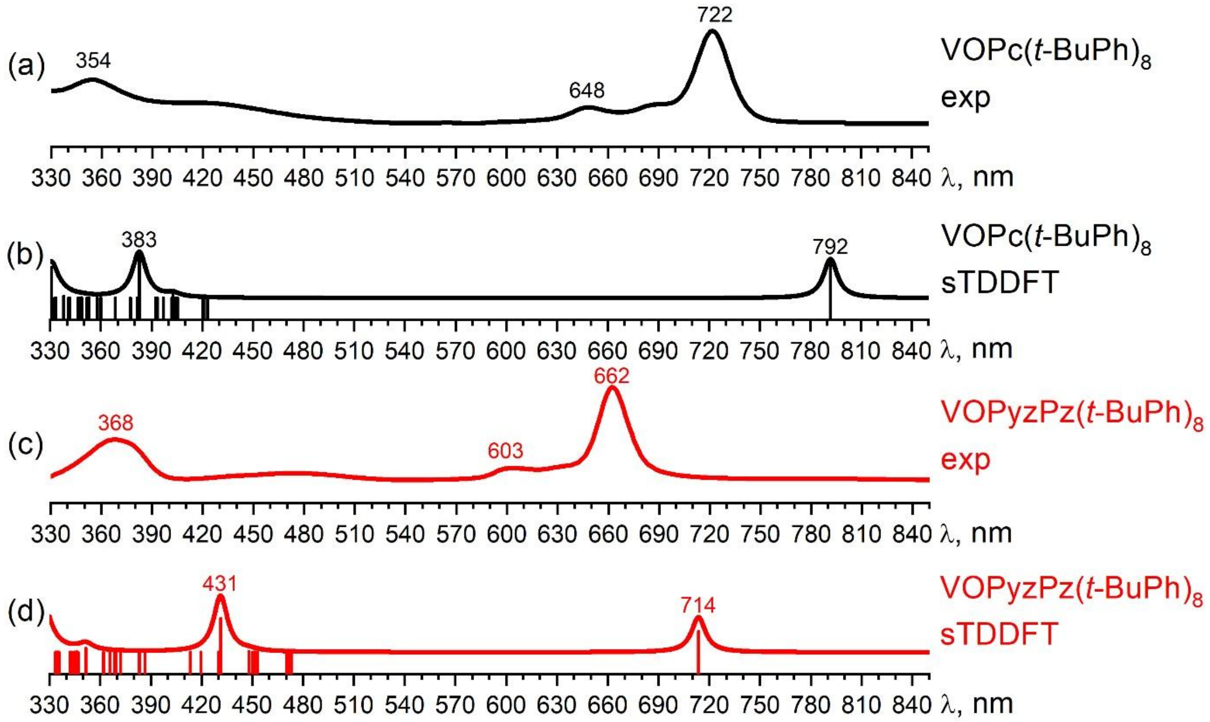

Figure S9. Experimental UV-Vis spectra for **VOPc(*t*-BuPh)$_8$** (a) and **VOTPyzPz(*t*-BuPh)$_8$** (c) in DCM in comparison with simulated (sTDDFT/CAM-B3LYP-D3BJ/def2-TZVP/CPCM:DCM // PBE0-D3BJ/def2-TZVP/gas) electronic absorption spectra for **VOPc(*t*-BuPh)$_8$** (b) and **VOTPyzPz(*t*-BuPh)$_8$** (d).

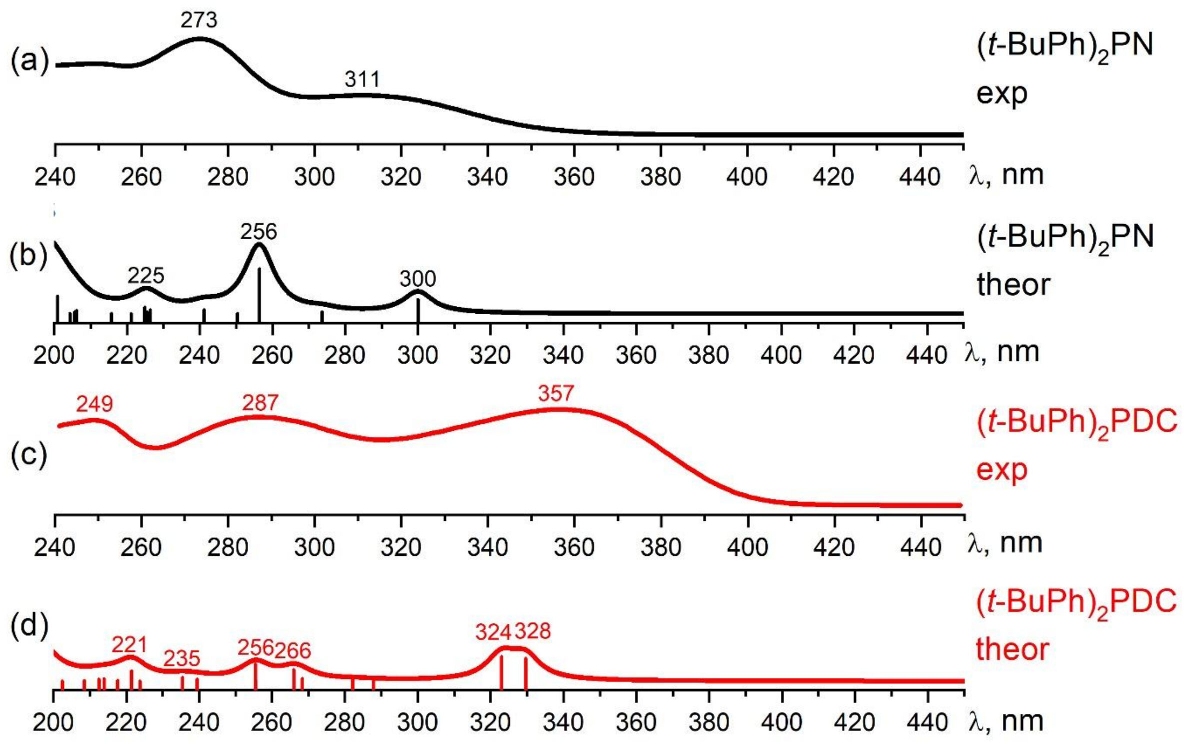

Figure S10. Experimental UV-Vis spectra for **(*t*-BuPh)$_2$PN** (a) and **(*t*-BuPh)$_2$PDC** (c) in DCM in comparison with simulated (CAM-B3LYP-D3BJ/def2-TZVP/CPCM:DCM // PBE0-D3BJ/def2-TZVP/gas) electronic absorption spectra for **(*t*-BuPh)$_2$PN** (b) and **(*t*-BuPh)$_2$PDC** (d).

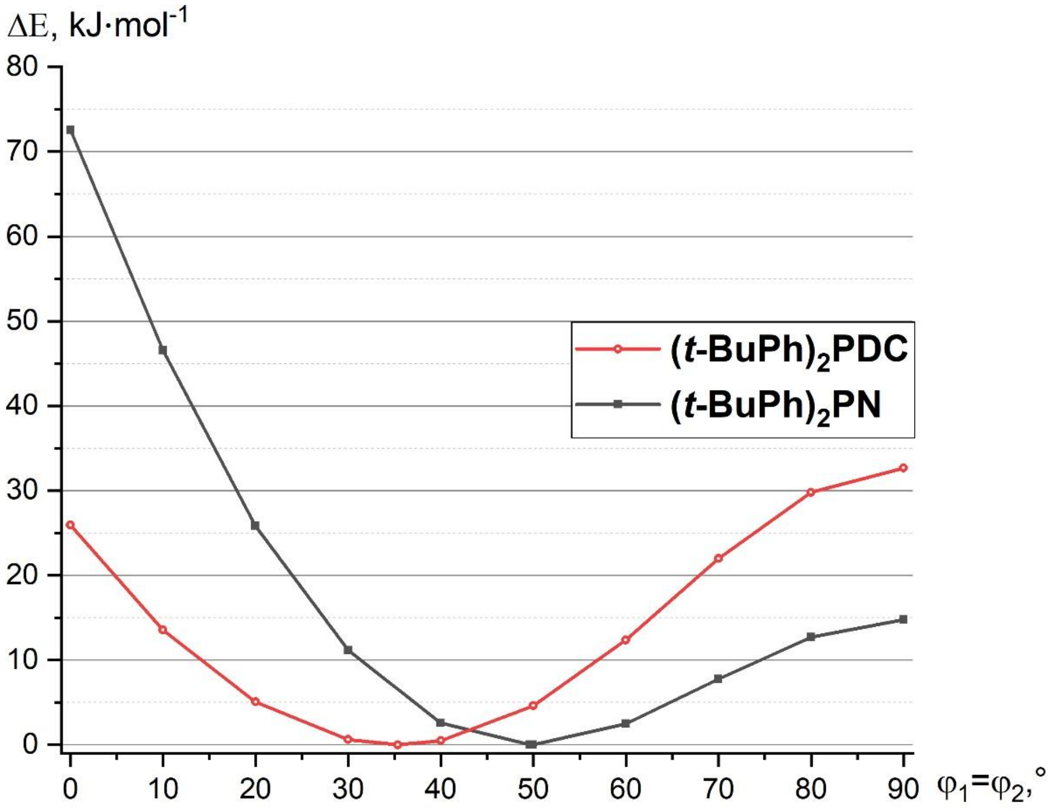

Figure S11. Relaxed potential energy function (PBE0-D3BJ/def2-SVP/gas) of internal symmetrical (φ_1=φ_2) rotation of two *t*-BuPh-groups in **(*t*-BuPh)$_2$PN** and **(*t*-BuPh)$_2$PDC** molecules. The calculations were carried out with two frozen dihedral angles φ_1=φ_2. For structures with φ_1=φ_2< φ_{opt}, there is a significant deviation of χ(C_5-X_1-C_6-C_1^{Ph}) from 180 ° due to the substantial steric repulsions caused by the close proximity of the phenylene rings.

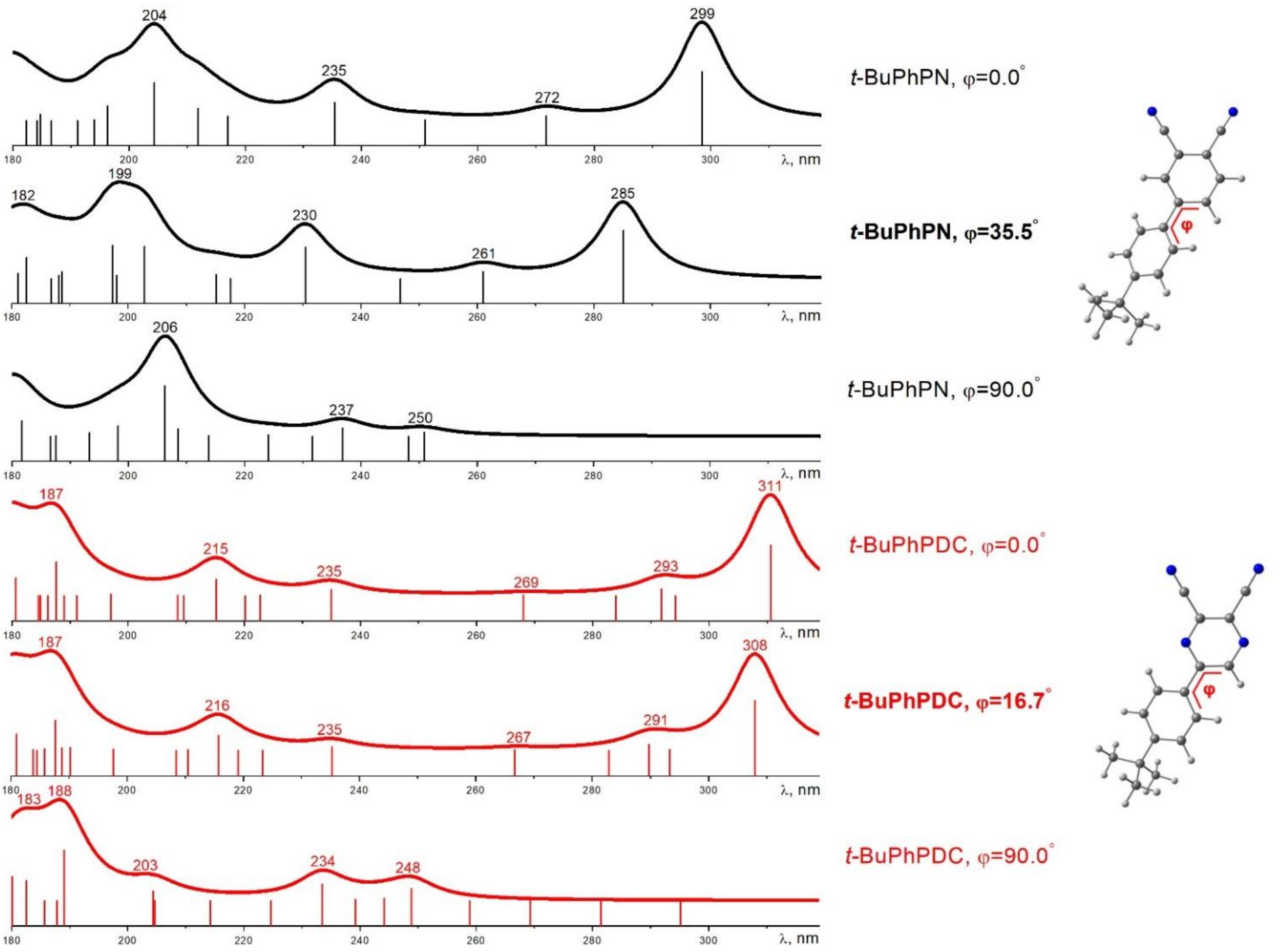

Figure S12. Normalized calculated TDDFT (CAM-B3LYP-D3BJ/def2-TZVP/CPCM:DCM // PBE0-D3BJ/def2-TZVP/gas) electronic absorption spectra for ***t*-BuPh-PN** and ***t*-BuPh-PDC** with different rotation angles φ. Structures of ***t*-BuPh-PN** and ***t*-BuPh-PDC** with φ=35.5° and 16.7° correspond to minima on the potential energy surfaces.

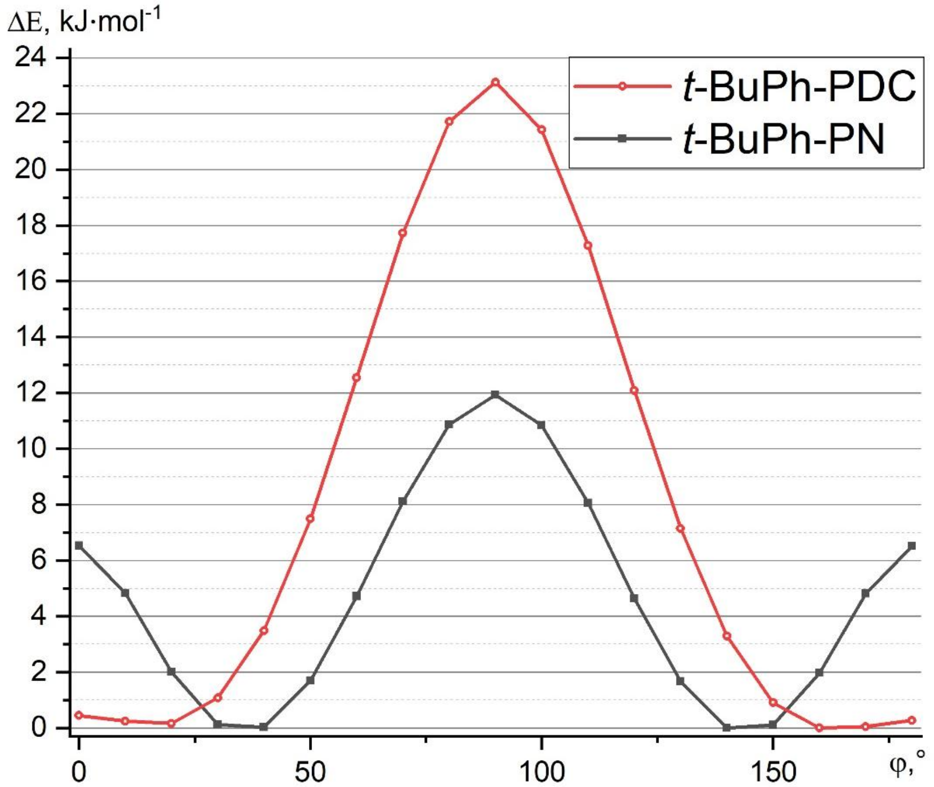

Figure S13. Relaxed potential energy function (PBE0-D3BJ/def2-TZVP/gas) of internal rotation of *t*-BuPh-group in ***t*-BuPhPN** and ***t*-BuPhPDC** molecules.

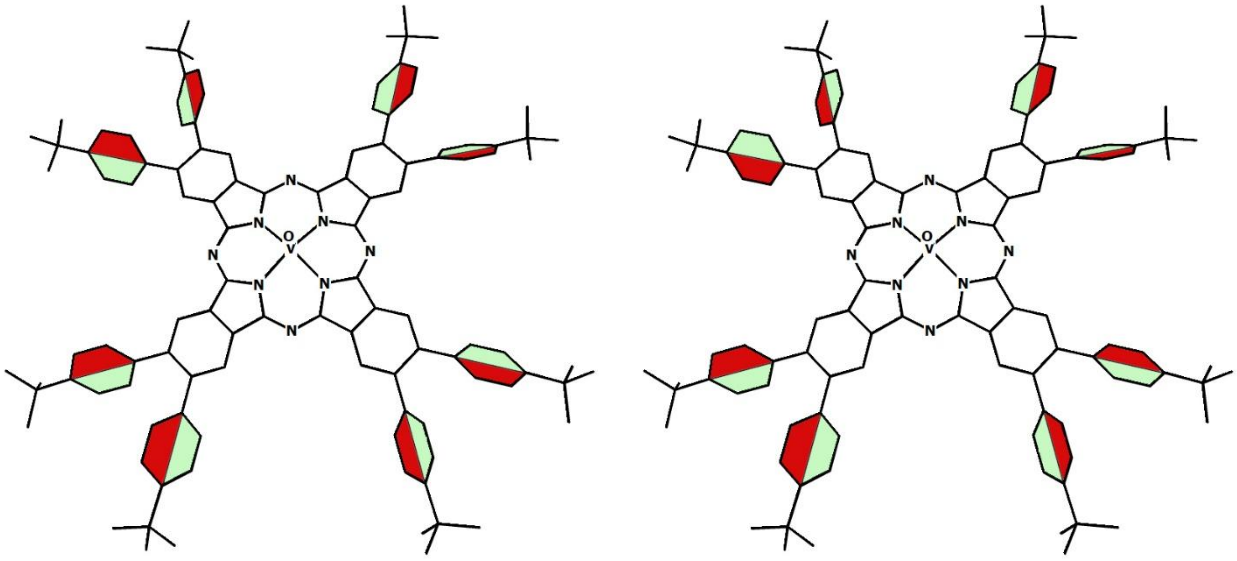

C_4 C_{2v}

Figure S14. Conformer models of **VOPc(*t*-BuPh)$_8$**: left – C_4-structure; right – C_{2v}-structure. The color shows the spatial arrangement of atoms relative to the planes of isoindole moieties: red – above the planes, green – under the planes.

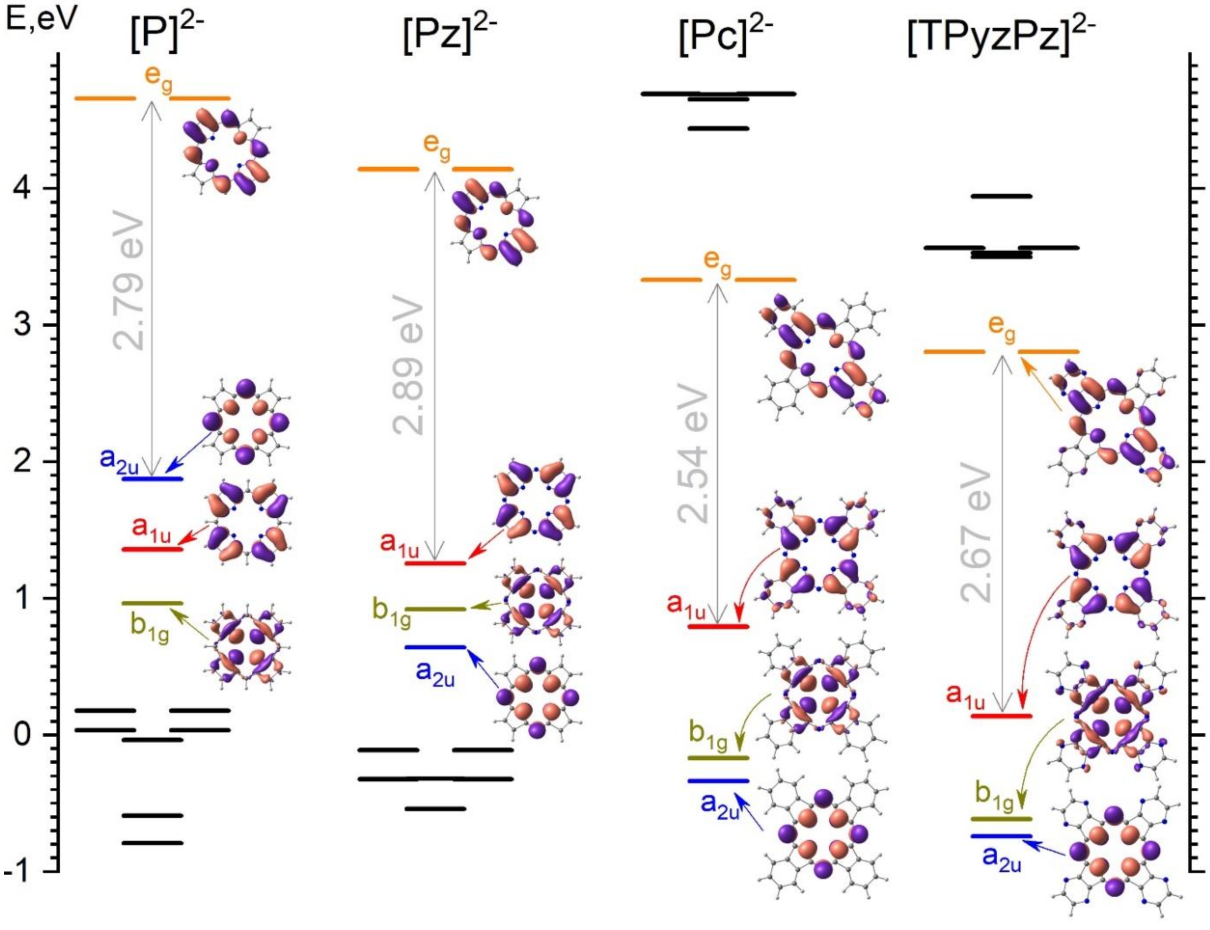

Figure S15. MO level diagram (ROPBE0-D3BJ/def2-TZVP/gas) and composition of frontier MOs for anions [porphyrin]$^{2-}$ - [**P**]$^{2-}$, [porphyrazine]$^{2-}$ - [**Pz**]$^{2-}$, [**Pc**]$^{2-}$ and [**TPyzPz**]$^{2-}$.

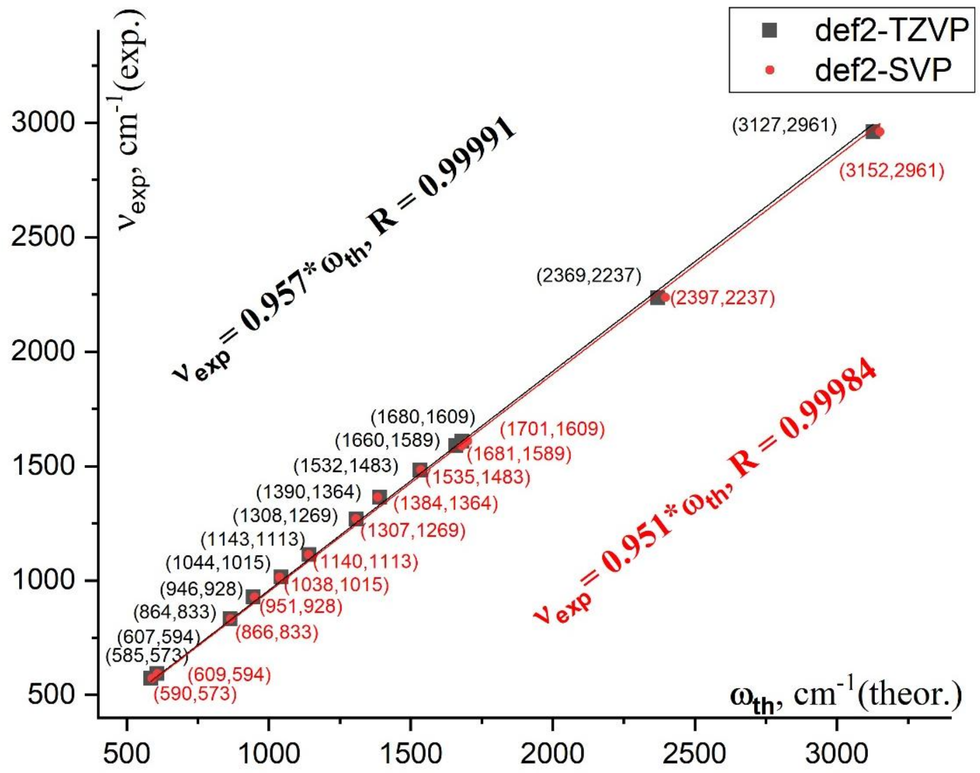

Figure S16. Correlation dependences $\nu_{exp} = f(\omega_{th})$: ν_{exp} and ω_{th} - the positions of the band maxima in the experimental and simulated (black squares ■ – PBE0-GD3BJ/def2-TZVP, Figure S3; red circles ● – PBE0-GD3BJ/def2-SVP, Figure S3) spectra for **(*t*-BuPh)$_2$PN**, respectively. R – adjusted R squared.

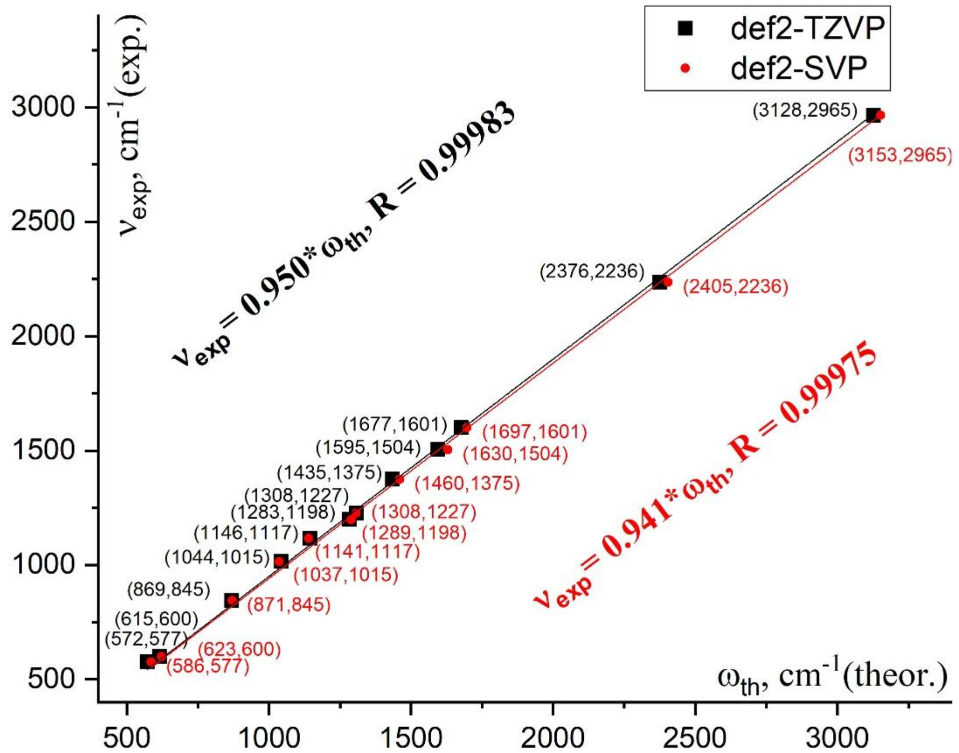

Figure S17. Correlation dependences $\nu_{exp} = f(\omega_{th})$: ν_{exp} and ω_{th} - the positions of the band maxima in the experimental and simulated (black squares ■ – PBE0-GD3BJ/def2-TZVP, Figure S4; red circles ● – PBE0-GD3BJ/def2-SVP, Figure S4) spectra for **(*t*-BuPh)$_2$PDC**, respectively. R – adjusted R squared.

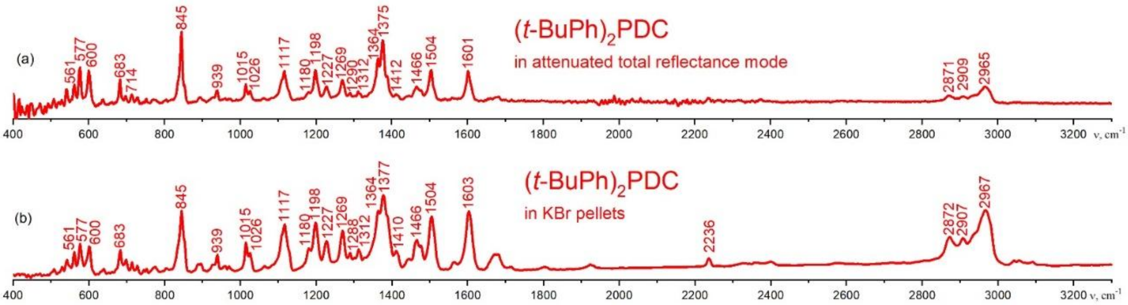

Figure S18. Experimental IR spectra for **VOPc(*t*-BuPh)$_8$**: (a) in attenuated total reflectance mode; (b) in KBr pellets.

Table S1. Calculated frequencies (ω_i), IR-intensities ($I_{IR\ i}$) and vibrational modes descriptions [a] for **(*t*-BuPh)$_2$PN.**

Sym	ω_i, cm^{-1}	0,957·ω_i, cm^{-1}	$I_{IR\ i}$, km·mol^{-1}	Assignment [a]	Our Exp.	[35]
B	586	560	16	γ(molecule): γ(Ph), γ(CN); δ(C-C-C);	573	569
B	607	581	19	δ(C-C-C); γ(Ph); γ(C-H)$_{Ph}$; ν(C-C);	594	594
B	865	827	43	γ(C-H)$_{Ph}$; γ(Ph);	833	835
B	946	905	13	γ(C-H)$_{PN}$;	928	915
B	948	908	3	δ(PN): δ(C_1-C_6-C_5); δ(C-C-C)$_{Ph}$; ν(C-C): ν(C-C)$_{Ph}$, ν(C_2-C^{CN}); γ(C-H)$_{PN}$;		
A	1043	998	8	δ(H-C-C)$_{Bu}$; ν(C-C): ν(C-C)$_{Bu}$; δ(Ph);	1015	1015
B	1045	1001	9	δ(H-C-C); δ(Ph); ν(C-C): ν(C-C)$_{Ph}$;		
A	1144	1094	24	δ(H-C-C); ν(C-C): ν(C-C)$_{Ph}$, ν(C_4^{Ph}-C_1^{Bu});	1113	1113
B	1307	1251	22	ν(C-C): ν(C_4^{Ph}-C_1^{Bu}), ν(C-C)$_{Bu}$; δ(H-C-C): δ(H-C-C)$_{Bu}$;	1269	1268
A	1308	1251	28	ν(C-C): ν(C_4^{Ph}-C_1^{Bu}), ν(C-C)$_{Bu}$; δ(H-C-C): δ(H-C-C)$_{Bu}$;		
B	1391	1331	24	δ(H-C-C)$_{Bu}$; ν(C-C);	1364	1363
B	1495	1431	15	δ(H-C-C)$_{Bu}$;	1462	1462
B	1498	1434	14	δ(H-C-C)$_{Bu}$;		
B	1513	1448	9	δ(H-C-C)$_{Bu}$;		
A	1513	1448	12	δ(H-C-C)$_{Bu}$;		
A	1532	1466	86	δ(H-C-C): δ(H-C-C)$_{PN}$, δ(H-C-C)$_{Ph}$; ν(C-C): ν(C-C)$_{PN}$;	1483	1484
B	1660	1588	37	ν(N-C)$_{PN}$; δ(H-C-C)$_{PN}$;	1589	1589
A	1680	1608	20	ν(C-C)$_{Ph}$; δ(H-C-C)$_{Ph}$;	1609	1608
A	2369	2267	45	ν(N^{CN}-C^{CN}); ν(C^{CN}-C_2);	2237	2233
B	2370	2269	14	ν(N^{CN}-C^{CN}); ν(C^{CN}-C_2);		
B	3045	2914	23	ν(C-H)$_{Bu}$;		
A	3045	2914	19	ν(C-H)$_{Bu}$;		
B	3046	2915	45	ν(C-H)$_{Bu}$;		
B	3051	2920	21	ν(C-H)$_{Bu}$;		
A	3051	2920	28	ν(C-H)$_{Bu}$;		
A	3127	2992	118	ν(C-H)$_{Bu}$;	2961	2964
B	3133	2998	69	ν(C-H)$_{Bu}$;		

[a] Based on PED. Coordinates are listed if their contributions are greater than ~10%. Coordinates are presented in descending order of their contributions. The designation "*Coord-1*: *Coord-2*, *Coord-3*;" means that the displacement along coordinates *Coord-2* and *Coord-3* are a part of the general displacement *Coord-1*. The following designations are used: ν(X-Y) – stretching of the X–Y bond; δ (A-B-C) – deformation of the valence angles A-B-C; γ – out-of-plane bending. Bu – butyl groups, Ph – phenylene group, PN – phthalonitrile moiety. Given that the molecule has the symmetry C_2, the following pairs of atoms are symmetrically equivalent: C_1 and C_4, C_2 and C_3, C_5 and C_6, etc. Therefore, it is assumed in assignment that, for example, vibration ν(C^{CN}-C_2) includes both vibration ν(C^{CN}-C_2) and ν(C^{CN}-C_3), etc.

Table S2. Calculated frequencies (ω_i), IR-intensities ($I_{IR\ i}$) and vibrational modes descriptions [a] for **(*t*-BuPh)$_2$PDC.**

Sym	ω_i , cm^{-1}	$0{,}950 \cdot \omega_i$, cm^{-1}	$I_{IR\ i}$, $km \cdot mol^{-1}$	Assignment [a]	Exp.
B	572	543	13	γ(molecule): γ(CN), γ(PDC), γ(Ph);	577
B	615	584	25	γ(CN); γ(Ph); γ(C-H)$_{Ph}$; ν(C-C); δ(C-C-C);	600
B	869	825	47	γ(C-H)$_{Ph}$; γ(Ph);	845
B	970	922	10	δ(PDC): δ(N_1-C_6-C_5); δ(C-C-C)$_{Ph}$; ν(C-C): ν(C-C)$_{Ph}$, ν(C_2-C^{CN});	939
A	1044	991	13	δ(H-C-C)$_{Bu}$; ν(C-C): ν(C-C)$_{Bu}$; δ(Ph);	1015
B	1047	994	10	δ(H-C-C); δ(Ph); ν(C-C): ν(C-C)^{Ph};	
A	1146	1089	38	δ(H-C-C); ν(C-C): ν(C-C)$_{Ph}$, ν(C_4^{Ph}-C_1^{Bu});	1117
A	1283	1219	84	Kekule: ν(C-C)$_{PDC}$, ν(N-C)$_{PDC}$;	1198
B	1308	1242	21	ν(C-C): ν(C_4^{Ph}-C_1^{Bu}), ν(C-C)$_{Bu}$; δ(H-C-C): δ(H-C-C)$_{Bu}$;	1227
A	1308	1242	24	ν(C-C): ν(C_4^{Ph}-C_1^{Bu}), ν(C-C)$_{Bu}$; δ(H-C-C): δ(H-C-C)$_{Bu}$;	
B	1391	1322	22	δ(H-C-C)$_{Bu}$; ν(C-C);	
A	1435	1364	271	ν(C-C): ν(C_6-C_1^{Ph}), ν(C_6-C_5); ν(N-C)$_{PDC}$; δ(H-C-C)$_{Ph}$;	1375
B	1443	1371	45	ν(C-C): ν(C_2^{Ph}-C_3^{Ph}), ν(C_5^{Ph}-C_6^{Ph}); δ(H-C-C)$_{Ph}$; ν(N-C)$_{PDC}$;	
B	1496	1421	13	δ(H-C-C)$_{Bu}$;	1466
B	1499	1424	14	δ(H-C-C)$_{Bu}$;	
B	1513	1438	12	δ(H-C-C)$_{Bu}$;	
A	1513	1438	22	δ(H-C-C)$_{Bu}$;	
B	1595	1515	139	ν(N-C)$_{PDC}$; ν(C-C);	1504
A	1677	1593	87	ν(C-C)$_{Ph}$; δ(H-C-C)$_{Ph}$;	1601
A	2374	2255	5	ν(N^{CN}-C^{CN}); ν(C^{CN}-C_2);	2236<br>2230 [36]
B	2378	2259	6	ν(N^{CN}-C^{CN}); ν(C^{CN}-C_2);	
B	3045	2893	19	ν(C-H)$_{Bu}$;	
A	3045	2893	21	ν(C-H)$_{Bu}$;	
B	3046	2894	44	ν(C-H)$_{Bu}$;	
B	3052	2899	23	ν(C-H)$_{Bu}$;	
A	3052	2899	26	ν(C-H)$_{Bu}$;	
A	3127	2971	107	ν(C-H)$_{Bu}$;	2965
B	3133	2977	68	ν(C-H)$_{Bu}$;	

[a] Based on PED. Coordinates are listed if their contributions are greater than ~10%. Coordinates are presented in descending order of their contributions. The designation "*Coord-1*: *Coord-2*, *Coord-3*;" means that the displacement along coordinates *Coord-2* and *Coord-3* are a part of the general displacement *Coord-1*.

The following designations are used: ν(X-Y) – stretching of the X–Y bond; δ (A-B-C) – deformation of the valence angles A-B-C; γ – out-of-plane bending. Bu – butyl groups, Ph – phenylene group, PDC – pyrazine-dicarbonitrile moiety. Given that the molecule has the symmetry C_2, the following pairs of atoms are symmetrically equivalent: N_1 and N_4, C_2 and C_3, C_5 and C_6, etc. Therefore, it is assumed in assignment that, for example, vibration ν(C^{CN}-C_2) includes both vibration ν(C^{CN}-C_2) and ν(C^{CN}-C_3), etc.
